# Mapping the Conformation Space of Wildtype and Mutant H-Ras with a Memetic, Cellular, and Multiscale Evolutionary Algorithm

**DOI:** 10.1371/journal.pcbi.1004470

**Published:** 2015-09-01

**Authors:** Rudy Clausen, Buyong Ma, Ruth Nussinov, Amarda Shehu

**Affiliations:** 1 Department of Computer Science, George Mason University, Fairfax, Virginia, United States of America; 2 Basic Science Program, Leidos Biomedical Research, Inc. Cancer and Inflammation Program, National Cancer Institute, Frederick, Maryland, United States of America; 3 Sackler Institute of Molecular Medicine, Department of Human Genetics and Molecular Medicine, Sackler School of Medicine, Tel Aviv University, Tel Aviv, Israel; 4 Department of Biongineering, George Mason University, Fairfax, Virginia, United States of America; 5 School of Systems Biology, George Mason University, Manassas, Virginia, United States of America; Stockholm University, SWEDEN

## Abstract

An important goal in molecular biology is to understand functional changes upon single-point mutations in proteins. Doing so through a detailed characterization of structure spaces and underlying energy landscapes is desirable but continues to challenge methods based on Molecular Dynamics. In this paper we propose a novel algorithm, SIfTER, which is based instead on stochastic optimization to circumvent the computational challenge of exploring the breadth of a protein’s structure space. SIfTER is a data-driven evolutionary algorithm, leveraging experimentally-available structures of wildtype and variant sequences of a protein to define a reduced search space from where to efficiently draw samples corresponding to novel structures not directly observed in the wet laboratory. The main advantage of SIfTER is its ability to rapidly generate conformational ensembles, thus allowing mapping and juxtaposing landscapes of variant sequences and relating observed differences to functional changes. We apply SIfTER to variant sequences of the H-Ras catalytic domain, due to the prominent role of the Ras protein in signaling pathways that control cell proliferation, its well-studied conformational switching, and abundance of documented mutations in several human tumors. Many Ras mutations are oncogenic, but detailed energy landscapes have not been reported until now. Analysis of SIfTER-computed energy landscapes for the wildtype and two oncogenic variants, G12V and Q61L, suggests that these mutations cause constitutive activation through two different mechanisms. G12V directly affects binding specificity while leaving the energy landscape largely unchanged, whereas Q61L has pronounced, starker effects on the landscape. An implementation of SIfTER is made available at http://www.cs.gmu.edu/~ashehu/?q=OurTools. We believe SIfTER is useful to the community to answer the question of how sequence mutations affect the function of a protein, when there is an abundance of experimental structures that can be exploited to reconstruct an energy landscape that would be computationally impractical to do via Molecular Dynamics.

## Author Summary

Important human diseases are linked to mutations in proteins. One such protein, Ras, undergoes mutations in over 25% of human cancers. Its biological activity involves switching between two distinct states, and several oncogenic mutations affect this switching. Despite significant investigation *in silico* via methods based on Molecular Dynamics, details are missing on how mutations affect the ability of Ras to access the states it needs to perform its biological activity. In this paper we present an algorithm that is capable of providing such details by exploring the breadth of the structure space of a given protein. The algorithm leverages information gathered in the wet laboratory on long-lived structures of the healthy/wildtype and mutated versions of a protein to effectively explore its structure space and reconstruct the underlying energy landscape. We apply this algorithm to the wildtype H-Ras and two known oncogenic variants, G12V and Q61L. Comparison of the energy landscapes elucidates the detailed mechanism by which the oncogenic mutations affect biological activity. We provide the algorithm for the research community to allow further investigation of the open question on how mutations to the sequence of a protein affect biological activity.

## Introduction

Mutations in protein sequences that lead to altered functions have been found to drive or participate in many human diseases [1, 2]. An important goal of molecular biology is to understand functional changes upon single-point mutations in proteins. This is a challenging task for both wet and dry laboratories. Investigations in the dry laboratory promise in principle to unravel the sequence-function relationship in proteins through a holistic, detailed characterization of a protein’s structure space and underlying energy landscape [3]. However, exploring the breadth of a protein’s structure space via MD-based conformational search algorithms remains computationally challenging [4].

In this paper we propose a novel conformational search algorithm, which is based on stochastic optimization rather than MD to circumvent the computational challenge of exploring the breadth of a protein’s structure space. We refer to this algorithm as SIfTER for **S**tructure **I**nitiated Search **f**or **T**ransient **E**nergy **R**egions. SIfTER exploits structural characterizations of a protein in the wet-laboratory to rapidly map the structure space and underlying energy landscape of a given protein sequence. By doing so, the algorithm allows mapping and juxtaposing landscapes of variant sequences of a protein and then relating observed differences to functional changes. Before relating further details on the novel algorithmic components that make this possible, we justify SIfTER in a gradual and systematic way on a hallmark case study in molecular biology, the family of Ras proteins.

Ras proteins mediate signaling pathways that control cell proliferation, growth, and development via guanine nucleotide-dependent conformational switching between an active and inactive structural state [5]. Ras is in its active (on) state when bound to GTP, and in the inactive (off) state when bound to GDP [5]. The rate of exchange between the GTP- and GDP-bound states is enhanced by two types of regulatory proteins, GTPase activating proteins (GAPs), which promote GTP hydrolysis, and guanine nucleotide exchange factors (GEFs), which promote GDP release, allowing for GTP to bind. Ras isoforms (H-, N-, and K-Ras are the most prevalent) exist, and they have unique physiological functions and roles in different human cancers and developmental diseases. Many structures have been reported and can be found in the Protein Data Bank (PDB) [6] for the wildtype (WT) ordered catalytic (G)-domain of H-Ras and several of its oncogenic variants.

The active (GTP-bound) and inactive (GDP-bound) states of the Ras catalytic domain differ structurally by 1.5Å. This change is concentrated near the nucleotide-binding site, which includes the switch regions SI (residues 25–40) and SII (residues 57–75) [7–15]. The structural change is driven by the formation of hydrogen bonds from the conserved residues T35 and G60 to the gamma-phosphate of GTP, which effectively closes the binding pocket [7]. When bound to GDP, and the gamma-phosphate is missing, the switch regions have fewer structural contacts to the ligand, and this allows the Ras catalytic domain to populate a more open structure [7, 9].

Mutations that deregulate Ras activity are found in over 25% of all human tumors [16]. In particular, two such mutations, G12V and Q61L, are shown to be oncogenic. The G12V mutation in H-Ras is implicated in bladder carcinoma [17, 18]. The Q61L mutation is implicated in melanoma due to its strongly reduced GTP hydrolysis in the presence of RAF-1 [19, 20]. NMR studies point to correlated conformational dynamics in Ras [21], which motivates further investigation of allosteric effects in the WT and variants. At present, our understanding of the impact of sequence variations on the ability of variants to populate functional conformations is limited to those structures documented in the PDB.

Seminal work by Grant and McCammon in 2009 projected the experimentally-probed conformation space of H-Ras onto two reaction coordinates extracted through a linear dimensionality reduction technique such as Principal Component Analysis (PCA) [7]. The two principal components (PCs) obtained from the PCA that captured most of the variance of the original structure data were used as reaction coordinates. The two-dimensional map of the conformation space of H-Ras exposed vast unpopulated regions by the WT and variants. Simple interpolation over existing structures in the two-dimensional embedding would not be accurate in delineating features of the energy landscape over the unpopulated regions of the conformation space (analysis in S8 Fig in the Supporting Information shows many regions of the energy landscape that are currently not covered by any known experimental structures of H-Ras). Moreover, many structural details would be sacrificed, as more coordinates or dimensions are needed to preserve the structural variance observed in the experimentally-probed conformation space.

More sophisticated conformational search algorithms, systematic or stochastic, are needed to handle more coordinates and explore the breadth of the conformation space. One could in principle devise a systematic search algorithm that imposes a grid over the specified coordinate axes. However, even at a small number of dimensions and a coarse resolution to define cells of the resulting grid, the number of structures needed to populated the grid would be prohibitive for any further energetic evaluation and improvement. Even at few cells per dimension and a modest number of dimensions, the number of structures easily reaches in the millions. As such, systematic grid-based searches have too high computational costs to be useful at all. Instead, either algorithms based on Molecular Dynamics (MD) or stochastic optimization remain viable.

Indeed, MD-based conformational search algorithms have been employed to study Ras structure and dynamics. MD-based simulations of Ras in uncomplexed and complexed forms were used in [22] to study subtle conformational and dynamics changes of Ras upon effector binding. A structural alphabet ensured removal of trivial roto-translations. Comparison of MD trajectories revealed changes due to downstream effector binding of Ras to Byr2, PI3K*γ*, PLC*ɛ*, and RalGDS [22].

The majority of MD-based approaches focus on mapping the conformation space of the uncomplexed form of Ras isoforms. The earliest such studies simulated local structural fluctuations around individual nucleotide states of uncomplexed Ras [23, 24]. Unbiased MD simulations in [25] captured spontaneous nucleotide-dependent transitions of the oncogenic H-Ras G12V variant [25]. Analysis of the uncovered regions of the energy landscape demonstrated that the energy barrier between the inactive and active states was lower in the H-Ras G12V variant than in the WT.

Due to the computational cost of unbiased MD simulations and demonstrated limitations in sampling, biased and accelerated MD simulations have been attempted, as well. Biased MD simulations resulted in unrealistic high-energy structures [26, 27]. On the other hand, accelerated MD, an approach originally proposed in [28], was shown to populate many regions of the H-Ras conformation space not observed in the wet laboratory [7]. Several known stable conformations of H-Ras variants were also found to be accessible to the WT. Multiple barrier-crossing trajectories were observed for the WT with 60ns-long accelerated MD simulations; as the authors noted, such trajectories would have been practically impossible to obtain with classical, unbiased MD simulations of the same length due to the high free energy barrier separating the active and inactive states in the H-Ras WT [7].

The sampling capability of accelerated MD was shown to greatly depend on the structure used to initiate a trajectory [7]. In several cases, accelerated MD simulations initiated from a WT inactive structure did not reach the crystallographic active structure, pointing to persistent limitations in sampling. Nonetheless, accelerated MD remains a viable option over classical MD and has been applied to characterize the dynamics of other Ras isoforms, several H-Ras variants [29], and has even been integrated in computational pipelines for identification of leads in drug design [30].

Non MD-based approaches devised to improve sampling over MD-based approaches [31] have been applied to study Ras, as well. For instance, work in [32] computed minimum-energy paths bridging between the active and inactive states through a modification of the conjugate peak refinement algorithm [33]. Other non MD-based approaches, such as CONCOORD [34], FIRST/FRODA [35, 36], and PEM [37–39] are designed to rapidly populate the conformation space in a neighborhood around a given structure. Though not directly applied to populate the conformation space of Ras, such methods could, in principle, if initiated from each existing crystallographic structure, provide a map of the conformation space of H-Ras. The foreseen difficulty would be on populating regions of the space with no experimentally-available structures in the vicinity.

Documented difficulties of MD-based methods and foreseen challenges with adapting existing non MD-based approaches motivate our proposal of SIfTER, a novel non MD-based conformational search algorithm capable of exploring the breadth of the structure space and mapping the underlying energy landscape of a protein. Since MD simulations remain computationally demanding and are challenged by complex high-dimensional search spaces [4], SIfTER implements stochastic optimization and yields a sample-based representation of the conformation space and energy landscape of a protein under investigation. We apply SIfTER to map and juxtapose energy landscapes of H-Ras WT and selected oncogenic variants to provide the energy landscape as the intermediate explanatory link between sequence mutations and functional changes.

SIfTER is a data-driven evolutionary algorithm. While in this paper we focus on the uncomplexed form of the catalytic domain of H-Ras in the WT form and two oncogenic variants, the algorithm is general. No system-specific information is exploited beyond experimentally-available structures of a protein. Unlike MD- and other non-MD based approaches that are limited to being initiated from a specific structure, SIfTER leverages information available in a collection of experimentally-determined structures documented for a protein. Inspired by the seminal work of Grant and McCammon in [7], SIfTER also employs PCA over experimentally-determined structures to define collective variables/parameters of the search space as well as effective ranges of these parameters. The algorithm efficiently draws samples from the resulting low-dimensional search space, and then maps samples through a novel multiscale procedure to all-atom conformations that are local minima in the all-atom energy landscape (the all-atom energy function used here is Rosetta *score12*). In this way, local minima in the energy landscape can be computed efficiently while still allowing for a wide search range within the space defined by the experimental structures.

As we demonstrate here, SIfTER reconstructs, for the first time, the all-atom energy landscapes of various sequences of the catalytic domain of H-Ras. The algorithm is able to efficiently do so by exploiting the existence of close to a hundred crystallographic structures of H-Ras WT and variants in the PDB. The guiding hypothesis for SIfTER is that documented structures of H-Ras WT and variants are also available and populated by a specific H-Ras sequence under investigation, though possibly with different population probabilities than in the native sequence probed in the wet laboratory. This is in essence the principle of conformational selection [40–43]. Grant and colleagues provided the first evidence of this by observing stable structures of variants populated by WT H-Ras [7]. This guiding principle allows treating the structures documented for WT and variants as possibly important locations in the energy landscape for a specific protein sequence under investigation.

Taken together, SIfTER produces an ensemble of all-atom conformations residing at local minima, effectively and efficiently providing a representation of the energy landscape relevant for understanding function. We juxtapose and analyze here in detail the landscapes obtained by SIfTER for WT H-Ras and two important oncogenic variants, G12V and Q61L. Our comparative analysis suggests that G12V and Q61L cause constitutive activation through two different mechanisms. G12V directly affects binding specificity while leaving the energy landscape unchanged, whereas Q61L has pronounced effects on the underlying landscape. In addition to validating existing biological knowledge, SIfTER provides for the first time a detailed view of the energy landscapes of H-Ras WT and variants and proposes novel structural states not observed in the wet laboratory. These structures provide the foundation for further structure-guided studies of function, molecular interactions, and therapeutics for oncogenic H-Ras variants. An implementation of SIfTER is made available to the community at http://www.cs.gmu.edu/~ashehu/?q=OurTools to encourage studies on the impact of sequence mutations on biological activity in other protein molecules.

## Results

86 structures satisfying various criteria, as detailed in the Materials and Methods section, are extracted from the PDB for H-Ras. 46 of these structures that reflect the state of the PDB up until 2009 are subjected to the PCA to obtain axes of search for SIfTER. These same structures were also analyzed in [25] via PCA and shown to span the range of structural displacements employed by H-Ras for its conformational switching between the active and inactive states. The rest of the 40 structures added to the PDB after 2009 were withheld by us from the PCA to constitute a validation set. An important part of our analysis below shows the ability of SIfTER to recover regions of the H-Ras conformation space containing the structures in the validation set.

**Fig 1:**
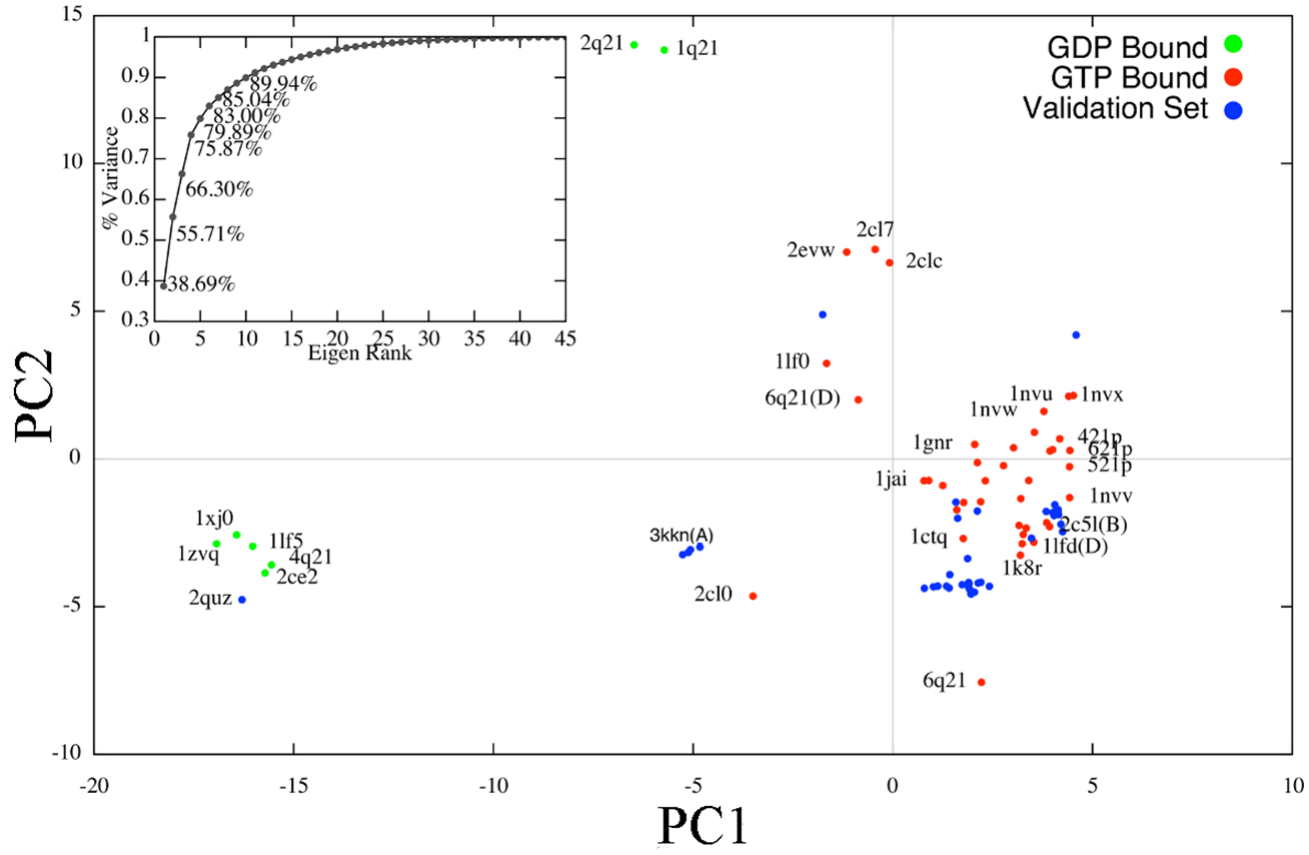
Projection of PDB-obtained Crystallographic Structures over Top Two PCs. Projections on the top two PCs are shown for all 86 collected structures of H-Ras. The 46 structures actually subjected to the PCA are in red (these correspond to the GTP-bound/inactive state) and in green (these correspond to the GDP-bound/active state). The 40 structures withheld from the PCA for validation purposes are shown in blue. The accumulation of variance subplot in the top left shows that PCA is effective for H-Ras. The 90% variance is achieved at 10 PCs. The two functional states of H-Ras are clearly separated by PC1. Projections of the 40 structures withheld from the PCA are contained in the same space.

Detailed analysis of the effectiveness of the PCA is provided in the Materials and Methods section, but Fig 1 summarizes this analysis by showing the projections of crystallographic structures on the top two axes/PCs obtained by the PCA. Fig 1 shows that the two functional (active and inactive) states are clearly separated, which is also in agreement with the results presented originally by McCammon and colleagues [7, 25]. In addition, a cumulative variance analysis detailed in the Materials and Methods section and summarized in the top left panel of Fig 1 indicates that only 10 PCs need to be specified as axes of search for SIfTER and yet retain 90% of the variance among the crystallographic structures. Moreover, only two PCs are needed to capture more than 50% of the variance; these two can be used to project SIfTER-obtained energy surfaces and visualize energy landscapes.

### Analysis of the Modes of Motion Captured by PCA

**Fig 2:**
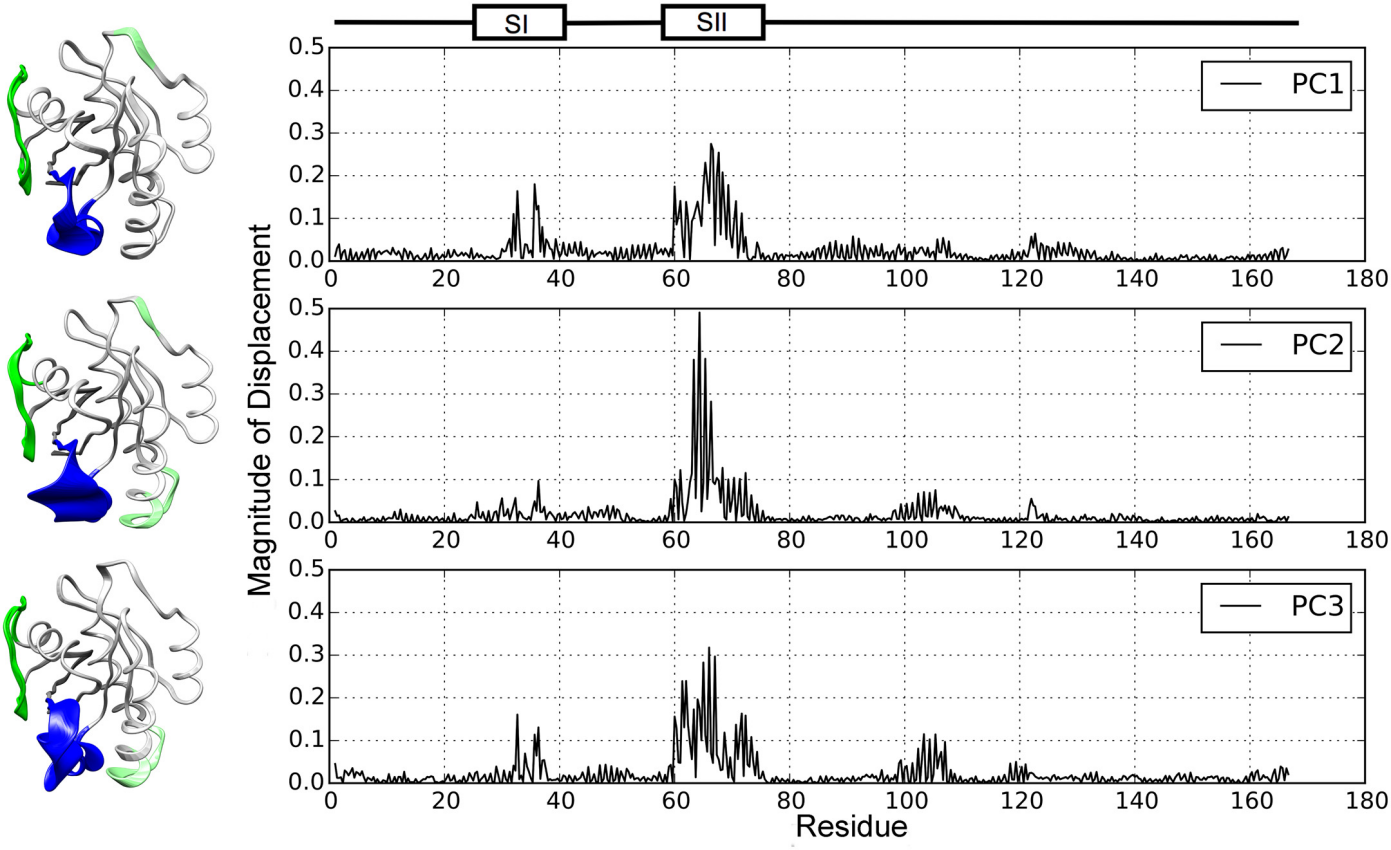
Structural Displacements Along Each of the Top Three PCs. Displacements of CAs along each of the three top PCs are visualized on the right by plotting the coordinates of each PC. The SI and SII regions are annotated to show that they undergo some of the largest internal fluctuations captured by PC1 and PC2. The displacements along each PC are visualized on the left on an H-Ras structure using Pymol [44]. The colored sections correspond to the switch regions of H-Ras, with SI in green and SII in blue. Sections colored in light green show regions with structural changes of a similar magnitude to the switch regions.

One can analyze in further detail the molecular motions associated with the top three PCs. Each PC is a vector containing 166 × 3 displacements for each of the *x*, *y*, *z* cartesian coordinates of the 166 CA atoms in the catalytic domain of H-Ras. These displacements are visualized in the right panel of Fig 2 by plotting the coordinates of each PC. The SI and SII regions are annotated. The displacements along each PC are additionally visually illustrated on an H-Ras structure in the left panel of Fig 2.

The right panel of Fig 2 shows that the region whose motions are captured consistently and are dominant along each of the top three PCs is the SII region ((amino acids at positions 57–75). The dominance of motions of the SII region has also been observed by Grant and McCammon in [7]. In particular, in [7], the helix *α*2 region (amino acids at positions 66 to 74) contained in SII is noted to be the major dynamic element of the Ras structure, in agreement with our observations here.

As Fig 2 shows, CA displacements in each of the top three PCs additionally capture the correlated motions between the SI (amino acids at positions 25–40) and SII regions. The regions that undergo the largest displacements are those of amino acids at positions 26 to 37, referred to as loop2 in the SI region, and those of amino acids at positions 66 to 74, the *α*2 helix in the SII region. The switch regions undergo the main structural changes in the GTP- to GDP- transition. Since PCA is capturing such deviations, this analysis lends further credibility to employing the reduced space of PCs as the search space for SIfTER to rapidly find more functional conformations of H-Ras. Moreover, since the top two PCs also account for over 55% of the variance (essentially allowing to capture 55% of the dynamics) and capture the structural changes between the GTP- and GDP-bound states, they are both effective to be employed in the structurization/grid by the local selection operator (detailed below) and to project the energy surface for the purpose of visualizing the energy landscape on 2 dimensions (projecting all SIfTER-obtained conformations on PC1 and PC2).

In addition, CA displacements in PC2 and PC3 show correlated motions that include amino acids at positions 93 to 110. This region is referred to as *α*3-loop7 in [7]. Taken together, the motions along PC1, PC2, and PC3 capture the dynamic linkage between three regions, SI (specifically, loop2 in SI), SII (specifically, *α*2 in SII), and *α*3-loop7. Such linkage has been observed previously in MD simulation studies [7]. In particular, the correlated motions between *α*2 and *α*3-loop7 have been previously noted to show a novel GTP-dependent correlated motion in Ras with functional implications [7]. These motions serve as a non-covalent communication route in Ras, and Grant and McCammon speculate that amino acids in these regions may be important for nucleotide-dependent modulation of membrane attachment and lateral segregation by linking the switching apparatus to the membrane interaction apparatus [7]. As noted by Grant and McCammon, while the loop3 region has been studied via mutations in the wet laboratory, the other regions, including the dynamic *α*3-loop7 region, though shown to undergo correlated motions in simulation, have received little attention in the wet laboratory. Our analysis seems to additionally emphasize the need for better understanding of the role of these regions in the function of Ras.

### Application of SIfTER on H-Ras WT and Variants

Using the top ten PCs as axes of the reduced search space, SIfTER is then applied to the WT, G12V, and Q61L sequences of H-Ras. It is worth noting that while the axes of the search space are the same for each application of SIfTER on each of the three sequences, the multiscale procedure that maps points sampled in the reduced search space to the space of all-atom conformations employs sequence information. Hence, the ensembles obtained by SIfTER on each application are different. On each application, the entire ensemble of all-atom conformations is stored. A conservative energy threshold of −100 Rosetta *score12* units is then applied in order to retain for further analysis only functional conformations (and essentially filter out false positives expected from any semi-empirical protein energy function). The determination of this threshold is not system-specific but is made based on the range of *score12* energy values obtained for crystallographic structures when their CA traces are threaded onto the WT sequence and then subjected to the multiscale procedure used by SIfTER. The range of resulting *score12* energies is observed to be from around −300 to around −100 units. Hence, only SIfTER-obtained conformations with energies no higher than −100 units are retained for further analysis.

The rest of our analysis below focuses first on the ability of SIfTER to recover functional conformations corresponding to crystallographic structures withheld from the PCA and then on visualization and comparison of energy landscapes constructed for each H-Ras sequence.

### Recovery of Known Functional Conformations

**Fig 3:**
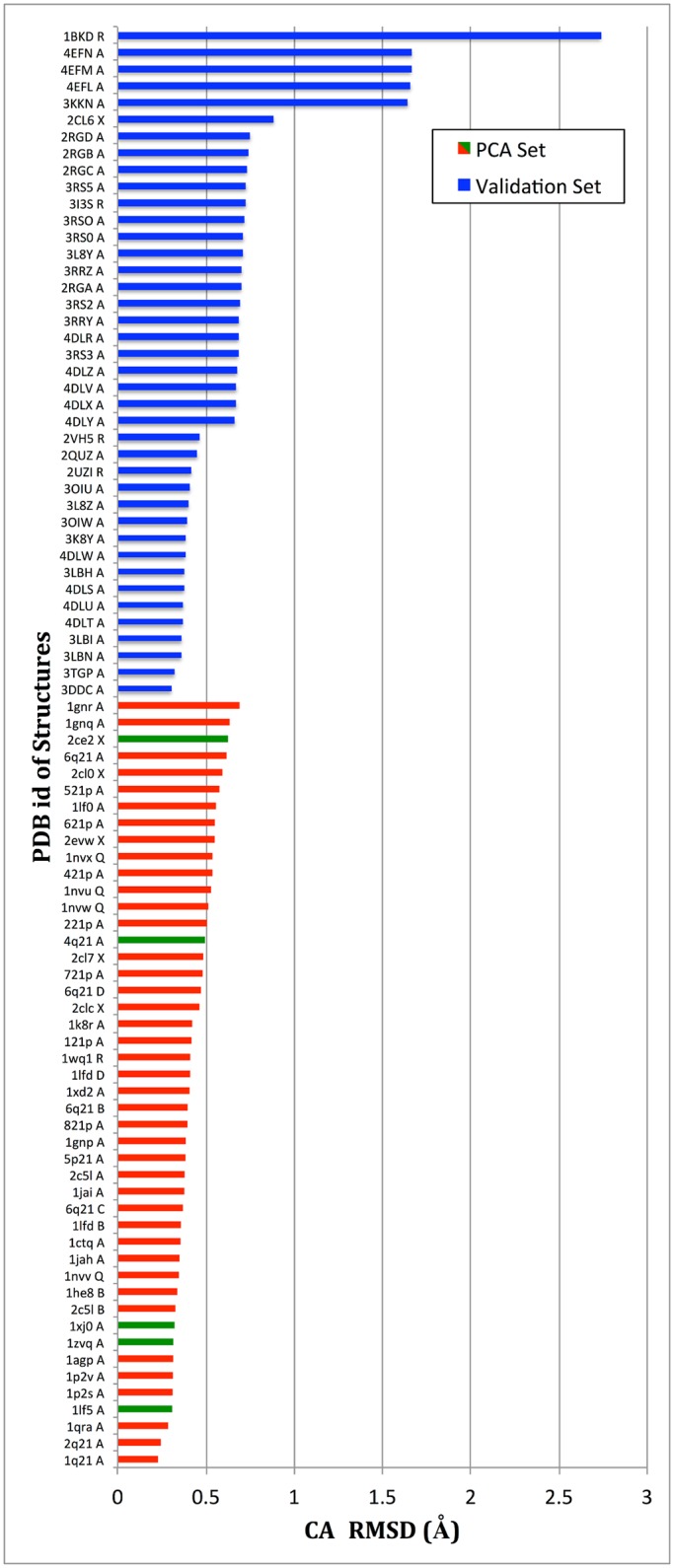
Reproduction of Crystallographic Structures Among SIfTER-generated Functional Conformations. Each crystallographic structure is compared to the sub-ensemble of functional conformations obtained by SIfTER, and the lowest CA RMSD is reported. CA RMSDs corresponding to GTP-bound structures are drawn in red, those corresponding to GDP-bound structures are drawn in green, and those corresponding to the 40 crystallographic structure withheld from the PCA for the purpose of validation are drawn in blue.

We validate first the capability of SIfTER to discover known functional conformations of H-Ras. We show data for the WT. For each of the 86 crystallographic structures, we find the closest conformation to that structure among the functional conformations obtained by SIfTER for WT H-Ras (all-atom conformations whose energies meet the energetic threshold described above). The distance between two conformations is measured through the well-known root-mean-squared-deviation (RMSD)after an optimal superimposition has been found that removes structural differences due to rigid-body motions [45]. In particular, Fig 3 shows CA RMSDs (the distribution of backbone RMSDs is very similar). It is not possible to report all-atom RMSDs, because many of these crystallographic structures may be on different sequences or have missing side-chain atoms even if reported for the WT sequence.

As can be seen in Fig 3, RMSDs for the structures withheld from PCA (in blue) are low, with the majority less than 1Å. As described in the Materials and Methods section on the presence of about 5 outlier structures with loop motions outside the SI regions, CA RMSDs higher than 1Å are only observed for a few outlier structures (details on these outlier structures are provided in S2 Table and S1 Fig in the Supporting Information). In comparison, the CA RMSDs for the 46 structures used by SIfTER to define the reduced space (in green and blue in Fig 3 to indicate structural state) are no higher than 0.7Å. Taken together, these results suggest that SIfTER is able to recover known functional conformations of H-Ras even though they are not directly incorporated in the algorithm.

### Mapping of Known Functional Conformations on the WT H-Ras Energy Landscape

**Fig 4:**
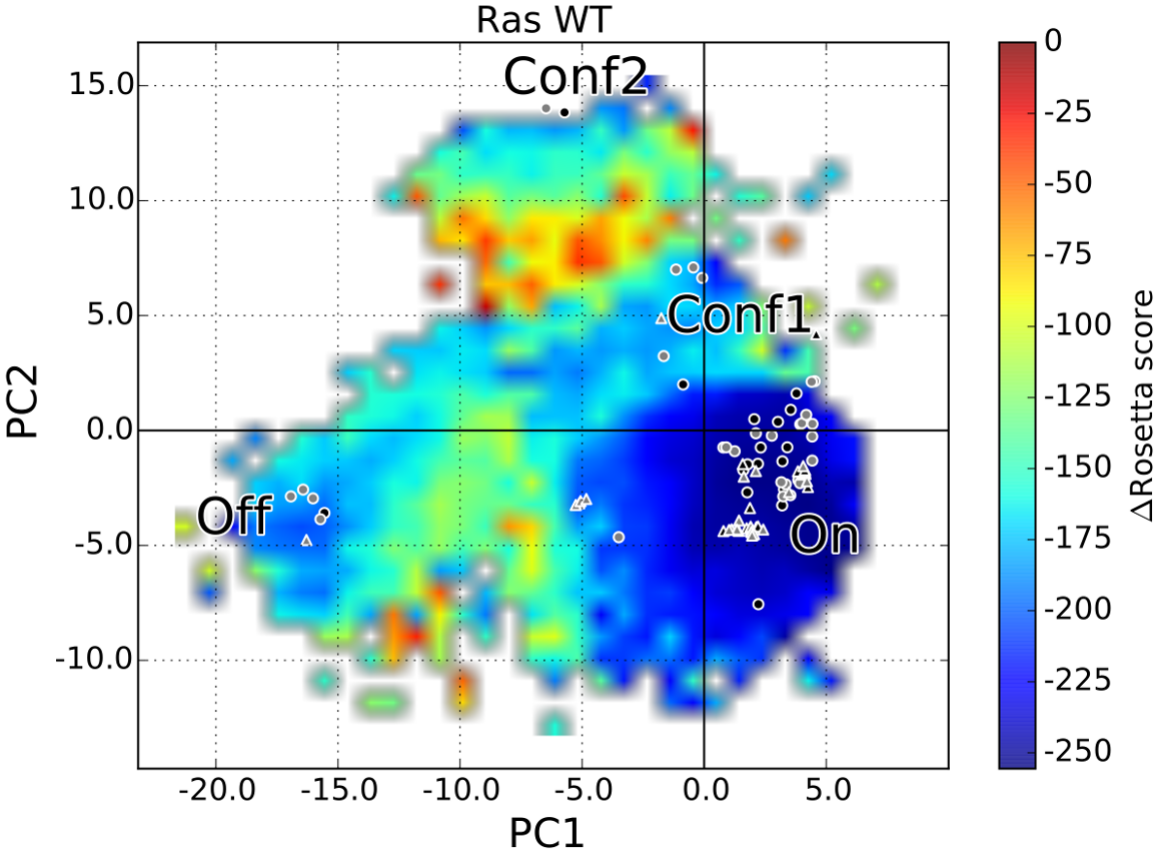
Mapping of Crystallographic Structures on SIfTER-obtained Energy Landscape for WT H-Ras. The energy landscape associated with functional conformations generated by SIfTER for WT H-Ras is shown here. All 86 crystallographic structures are projected onto the top two PCs to mark their locations on the landscape. The 46 structures used by SIfTER to define the reduced search space and obtain PCs are drawn as circles. The 40 structures withheld from SIfTER for the purpose of validation are drawn as triangles. Crystallographic structures reported for the WT sequence are filled in black, whereas those reported for other variants are filled in gray.

In addition to being able to recover known functional conformations, SIfTER also provides the ability to map the location of these conformations on the energy landscape. Fig 4 shows the energy landscape associated with functional conformations generated by SIfTER for WT H-Ras. The landscape is a projection of the energy surface over the top two PCs for the purpose of visualization. This two-dimensional projection of the space of functional conformations is color-coded as follows. A grid is laid over the embedding, with cells of size 1. Each cell is then colored by the median energy score of the conformations with projections in that cell. The bilinear interpolation in the *imshow* python utility is employed for this purpose. For ease, the color bar shows not the range of absolute *score12* energy values but instead the difference from the lowest-energy value. Fig 4 additionally shows the locations of all collected 86 crystallographic structures on the SIfTER-obtained energy landscape for WT H-Ras. A crystallographic structure can be easily projected onto given PCs, as described in detail in the Materials and Methods section. Projections of the 46 structures used by SIfTER to define the reduced search space are annotated differently from projections of the unused set of 40 structures. Moreover, crystallographic structures reported for the WT are specially annotated.

Fig 4 allows visualizing the location of experimentally-obtained structures onto the energy landscape. Several observations can be made. The majority of crystallographic structures reported for the WT are all on regions of the landscape that correspond to the deepest basins (darker blue regions). This applies to both structures employed by SIfTER to define the search space and those withheld for the purpose of validation. Structures reported for variant sequences also map to low-energy regions, which supports the hypothesis that these structures, though not reported for the WT, can be used as representatives of meta-stable states to guide a data-driven algorithm like SIfTER. In addition, no crystallographic structures, whether reported for the WT or variant sequences, are found to lie on energy barriers. This further lends credibility to SIfTER’s ability to find novel features of the energy landscape that a simple interpolation of energies in the PC1-PC2 embedding or limited exploration around a structure cannot produce. The energy landscape elucidated by SIfTER allows better understanding the menu of functional conformations used by H-Ras WT, beyond the space probed directly in the wet laboratory.

### Comparison and Analysis of SIfTER-obtained Energy Landscapes of WT and Variant Ras

The rest of our analysis focuses on novel knowledge that SIfTER confers about the three sequences of H-Ras studied in this paper by comparing energy landscapes generated by SIfTER for each sequence. We recall that the landscapes are projections of functional conformations generated by SIfTER on each sequence onto the top two PCs. Color-coding of the two-dimensional embeddings is as described above. In addition, the analysis below provides not only Rosetta *score12* landscapes, but also Amber *ff12SB* landscapes. The latter are obtained by subjecting functional conformations to a short energy minimization protocol in AMBER, described in detail in the Materials and Methods section.

**Fig 5:**
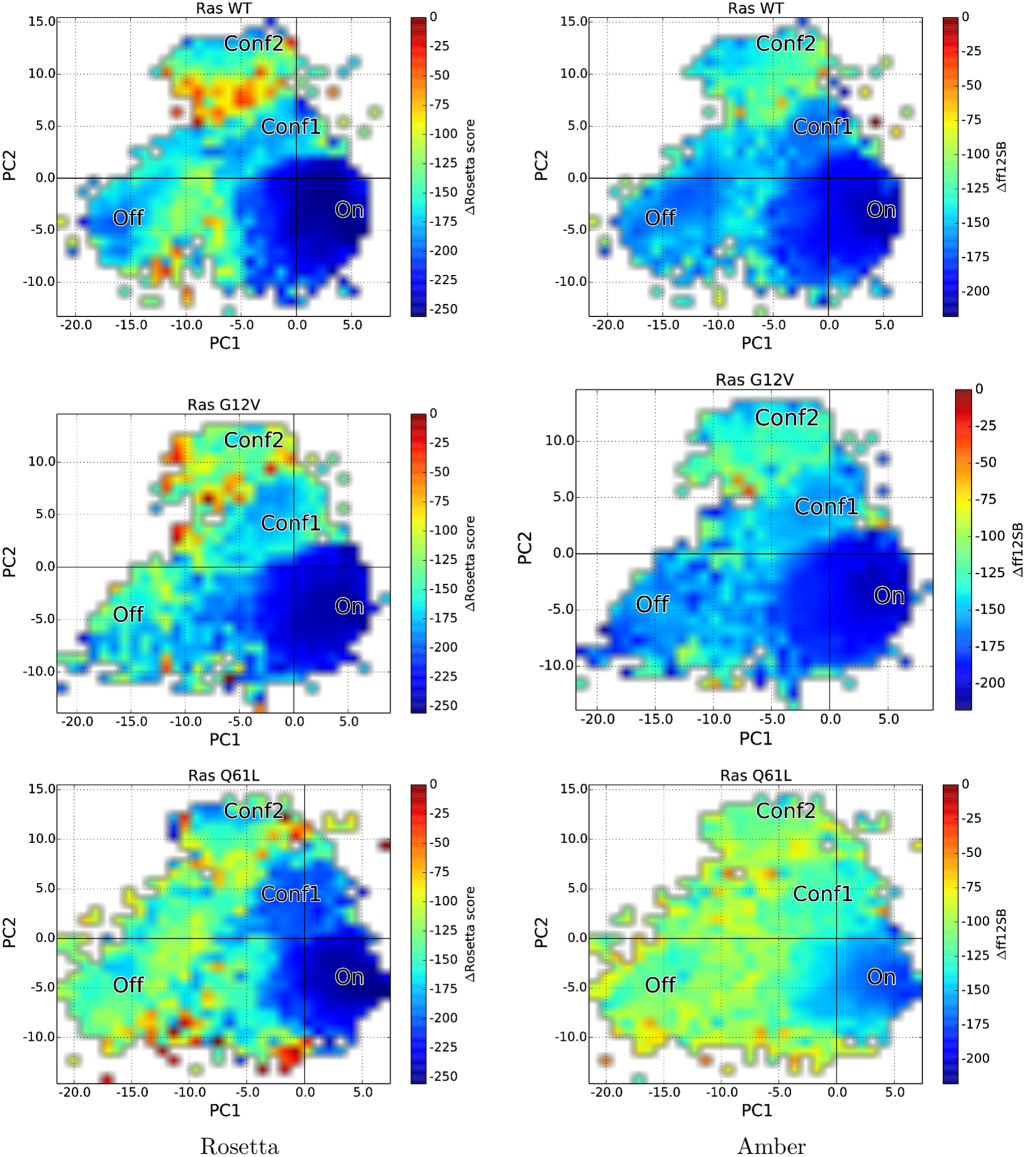
Comparison of Energy Landscapes Obtained by SIfTER for each H-Ras sequence. Obtained Rosetta and AMBER energy landscapes are shown for WT, G12V, and Q61L H-Ras. The location of the inactive and active structural states of H-Ras are indicated by the respective *On* and *Off* annotations. Other structural states corresponding to novel, observed basins in the landscapes are annotated by *Conf1* and *Conf2*.

The Rosetta and Amber landscapes for each H-Ras sequence studied here are shown in Fig 5. The left column shows the Rosetta *score12* landscapes, and the right column shows the AMBER *ff12SB* landscapes. The top row shows the landscapes obtained for the WT, the middle row shows the landscapes obtained for the G12V variant, and the bottom row shows the landscapes obtained for the Q61L variant. We note that the color bars do not show absolute energy values, but differences from the lowest-energy value obtained for each sequence. This allows focusing on relative scales rather than absolute energy values, which can be different among energy functions.

The Rosetta and AMBER SIfTER-obtained energy landscapes agree very well for the WT sequence (see top row in Fig 5). Four basins are observed, annotated *On*, *Conf1*, *Conf2*, and *Off*. The *On* basin is designated as such based on the location of projections of GTP-bound structures on the PC1-PC2 map (shown in Fig 1). Similarly, the *Off* basin is designated as such based on the location of projections of GDP-bound structures on the PC1-PC2 map. It is reassuring to note that the *On* and *Off* structural states both correspond to deep basins (blue regions) in the Rosetta and AMBER energy landscapes generated by SIfTER for the WT and G12V H-Ras. However, for both sequences, the GTP-bound/active structural state resides in a deeper basin than the GDP-bound/inactive structural state. This difference is starker on the Rosetta landscapes for each of the sequences.

Two novel higher-energy basins, annotated *Conf1* and *Conf2*, are additionally observed. Comparison with the projections of crystallographic structures in Fig 1 reveals that the *Conf2* basin corresponds to the same location as structures with PDB ids *2q21* and *1q21*. These structures are described in a study on the G12V mutation [46] but have not been reported as possibly functional conformations of the WT H-Ras. The energy landscape analysis here suggests that these structures may be functional, from a thermodynamic availability point of view, but perhaps difficult to access for the WT. The reason for this is that the *Conf2* basin is surrounded by high-energy barriers that may prevent the WT sequence from readily adopting this alternative functional state.

The other, novel *Conf1* basin corresponds to an unanticipated structural state. The crystallographic structures whose locations in the PC1-PC2 map correspond to this basin are those with PDB ids *1lf0* and *6q21(D)*. The structure with PDB id *1lf0* is the crystallographic structure of H-Ras A59G variant in the GTP-bound state [47]. This variant adopts a conformation that is an intermediate between the GTP- and GDP-bound states of WT H-Ras. Prior work has noted the intermediate nature of A59G conformations, as removing the gamma-phosphate of the bound GTP from the structure of A59G led to a spontaneous GTP-to-GDP conformational transition in a 20-ns unbiased MD simulation [29]. The location of the *Conf1* basin found by SIfTER confirms this and sheds additional novel insight. The experimentally-probed structure for A59G H-Ras (PDB id *1lf0*) can indeed be populated by WT H-Ras as a semi-stable structural state. The corresponding *Conf1* basin may indeed mediate the transition between the *On* and *Off* basins. This may be a general mechanism for the WT and the two oncogenic variants studied here. No high-energy barriers are noted for this possible transition in the landscapes obtained for the WT and G12V sequences (and to some extent, the Q61L sequence, as well, though there are starker differences between the Rosetta and AMBER landscapes for the Q61L variant).

The observations made above for the alignment of known and novel structural states with basins in the landscapes obtained for WT H-Ras largely transfer to observations for the G12V variant (see middle row in Fig 5). The AMBER landscape depicts basins *Conf1* and *Conf2* as being deeper than in the Rosetta landscape. Comparing the Rosetta landscape for the G12V sequence to the WT landscape shows that the *Off* basin has also become less defined in the G12V variant. In particular, in the AMBER landscape for G12V, the barrier between the *On* and *Off* basins has been significantly reduced. This is the main change between the WT and G12V H-Ras landscapes. The reduced stability of the GDP-bound state for the G12V variant suggest that it may be this change that contributes to the oncogenic activity associated with the G12V mutation. However, the change in the G12V energy landscape is small, which may further suggest that a change in binding specificity due to the proximity of G12V to the binding site may also contribute to the oncogenic activity.

These findings agree with published computational and experimental studies on the G12V variant. In particular, previous MD simulation studies have shown that both GTP-bound and nucleotide-free G12V H-Ras sample a wide region of conformation space, indicating the absence of significant changes in the conformation space due to the G12V mutation [25]. Experimentally, it has been shown that the G12V variant has similar binding affinity of ATP as the WT, though the V12 side chain in the G12V variant hinders correct orientation of water molecule needed for ATP hydrolysis [48]. The bulky V12 side chain in the G12V variant is thought to lower the GTPase activity through a steric interference over this catalytic process [49].

The Rosetta and AMBER SIfTER-obtained energy landscapes for the Q61L variant agree on the main features (see bottom row in Fig 5). In both landscapes, the *Conf2* and *Off* basins have all but disappeared. While the *Conf1* basin is retained in the Rosetta landscape, and the On basin extends towards the Conf1 basin, the Conf1 basin disappears in the AMBER landscape. Both the Rosetta and AMBER landscapes agree that mainly the *On* basin is retained, which corresponds to the GTP-bound state. This suggests that the oncogenic mutation Q61L causes significant changes to protein stability by causing the protein to become much more rigid, thereby destabilizing all structural states except the GTP-bound state associated with the *On* basin. By essentially only allowing Q61L to adopt the GTP-bound state, this mutation causes H-Ras to be constitutively activated, which may initiate the cascade of cellular processes resulting in unregulated cell growth and cancer. We point out that early studies through classical MD simulations succeeded in capturing the active to inactive transition in Q61L largely because of an observed lower free-energy barrier compared to the WT [7]. This is in agreement with our observations, and the detailed energy landscapes obtained here for the Q61L variant provide an easy visualization of why this is the case for the first time.

In addition, our findings on the rigidification of the GTP-bound state in the Q61L variant have been corroborated in the wet laboratory [19, 20]. In particular, work in [50] shows that Q61L is not able to hydrolyze GTP in the presence of Raf and thus is a constitutive activator of this mitogenic pathway. In addition, the study shows that the newly-resolved crystal structures of the Ras-GppNHp/Raf-RBD and RasQ61L-GppNHp/Raf-RBD complexes, in combination with MD simulations, exhibit a rigid SII relative to the WT.

Finally, it is worth noting that one of reasons the AMBER landscapes are morphologically very similar to the Rosetta landscapes is that the AMBER minimization of each structure obtained by SIfTER does not introduce significant structural changes, particularly to the positions of the CA atoms. This is shown in the Supporting Information in S5 Fig. Since the PCs are over CA traces, no significant morphological changes are expected. However, as the analysis above has demonstrated, changes in the relative depths of various regions on the landscape are expected, due to the employment of a different energy function.

For completion, projections of the landscape along PC3 are also provided and can be found in S9 Fig in the Supporting Information document. Projections along PC3 fail to provide any more separation of the basins identified above, as expected, given that variance along PC3 is minimal compared to PC1 and PC2; in other words, the dynamics of H-Ras can largely be accounted for by PC1 and PC2. In addition, while the above analysis color codes the cells of the PC1-PC2 maps by the median energy of structures mapping to a cell, S10 Fig in the Supporting Information document color codes by variance. S10 Fig shows lower variance for the four identified structural states/basins; this is expected, as SIfTER is a stochastic optimization algorithm that explores lower-energy regions in greater structural detail.

### Detailed Look into Conformations Representative of Captured Basins

**Fig 6:**
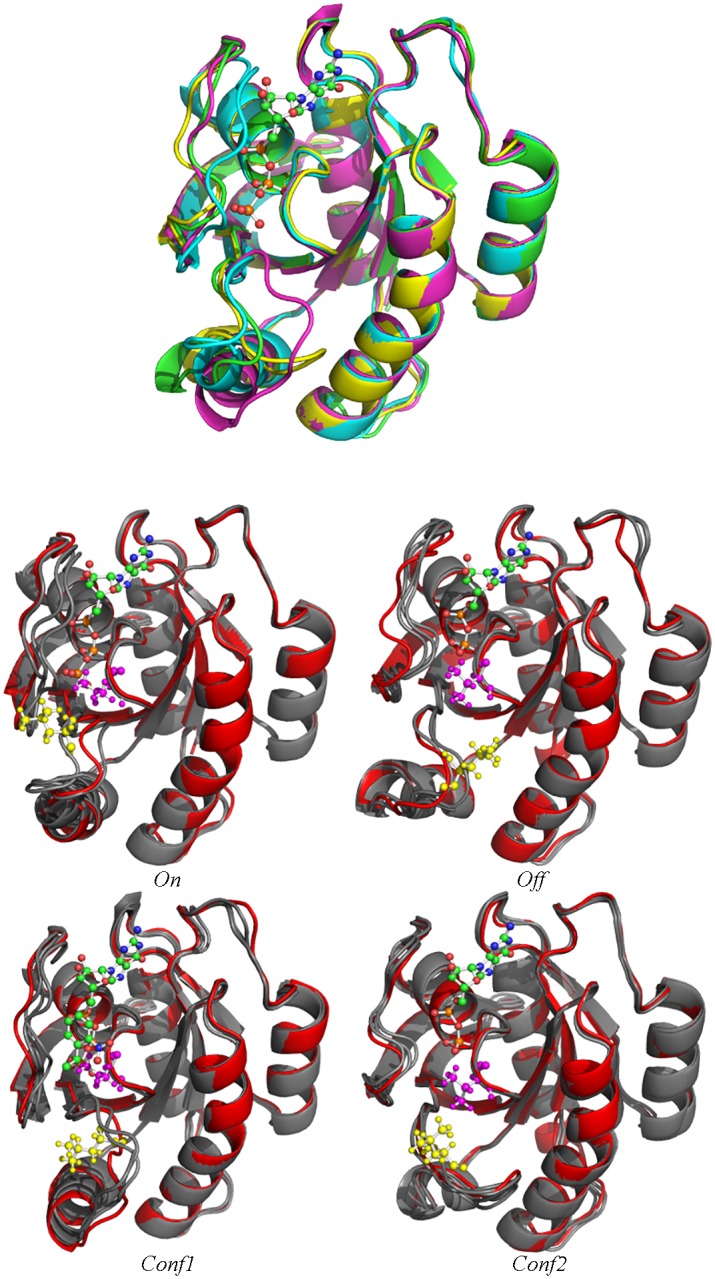
Structural States Corresponding to SIfTER-obtained Basins. Top panel: A representative conformation is drawn from each of the four basins obtained for the WT H-Ras by SIfTER. Different colors are used to distinguish conformations and see the breadth of the structural change captured by the four basins corresponding to the stable and semi-stable states. Yellow corresponds to the *Conf2* basin, purple to the *Conf1* basin, green to the *Off* basin, and blue to the *On* basin. Conformations are drawn in ribbon representation. The GTP/GDP ligand is also shown, drawn in a ball-and-stick representation. Bottom panel: Conformations representative of a given basin in each of the three sequences are superimposed and drawn in gray. A crystallographic structure projecting to each of the four basins is also drawn (in red). Conformations are drawn in ribbon representation. The side chain of V12 is drawn in purple and that of L61 in yellow in ball-and-stick representation. The GTP/GDP ligand is also drawn in ball-and-stick representation. Pymol [44] is used for rendering.

The structural states corresponding to each of the 4 basins recovered by SIfTER for WT H-Ras are shown in Fig 6 (top panel). 4 conformations representing each of these 4 basins are superimposed over one another. Superimposition of these conformations allows visualizing the slight structural changes associated with the four different structural states found by SIfTER.

Another visualization of each of the four structural states corresponding to the four basins is provided in Fig 6 (bottom panel), which now shows these states not only for WT H-Ras but the other two variants, as well. Crystallographic structures mapping to the four basins are also shown for reference.

Fig 6 demonstrates that conformations taken from the same region in the landscape, regardless of which sequence, have the same conformational topology. In addition, crystallographic structures mapping to the same region (shown in red) also have very similar topology. In addition, Fig 6 shows that the G12V mutation is very close to the binding site for the ligand. This supports the conclusion that the G12V mutation derives some of its oncogenic properties due to the mutation interacting with the ligand. On the other hand, the Q61L mutation is located much further away from the binding site, but position 61 is part of the SII region. As we have shown previously, this is the region of H-Ras that undergoes the largest conformational change between the GTP- and GDP-bound states. Taken together, these observations support the argument that the Q61L mutation has major effects on the stability of H-Ras by effectively rendering the GDP-bound state inaccessible.

## Discussion

The energy landscapes obtained by SIfTER offer the most comprehensive energetic analysis of H-Ras thus far available. For the first time, energy landscapes have now been reported for the WT and two oncogenic variants of H-Ras. The energy landscapes connect individual experimentally-known conformations to their relative energies in the global scale. Most importantly, some of these conformations are located on the energy barrier connecting active and inactive conformations, thus providing important insight into Ras function and dynamics. In addition, semi-stable structural states (corresponding to the Conf1 and Conf2 basins) are revealed for WT H-Ras. Mechanistic insight is obtained for a possible Conf1-mediated transition between the On and Off states. Juxtaposition of the energy landscapes reveals a thermodynamic argument for changes to function in the two oncogenic variants G12V and Q61L.

Computed energy landscapes are potential energy landscapes without entropic effects. However, entropic effects are implied visible in the width of the discovered basins. Regarding entropic effects, flexible structures in shallow basins should have higher entropy and so can be further stabilized. Deep basins separated by high energy barriers may have unfavorable entropic effects. For instance, the Q61L mutation causes a higher-energy barrier between different conformational states and rigidifies H-Ras, which also has entropic implications.

It is also worth noting that what we have studied here is the “intrinsic” energy landscape without the effects of the GTP/GDP nucleotides. When GTP/GDP bind to Ras, the relative energies in the energy landscape change; the gamma phosphate of GTP can further stabilize the closed GTP conformation. The essence of the conformation selection and population theory is that these conformations pre-exist prior to the ligand (GTP/GDP) binding, which is what we reveal in this paper.

Application of SIfTER to the G12V H-Ras variant reveals that the G12V mutation has a small effect on the energy landscape. Analysis of the landscape and location of the mutation relative to the GTP/GDP binding site suggest that the oncogenic properties of this mutation may result from a combination of altered protein stability and changed binding specificity. The Q61L mutation has a more profound effect, essentially rigidifying H-Ras to the GTP-bound state. Mutation-induced structural changes, such as the rigidification of the Q61L variant, affect the intrinsic GTP hydrolysis activity in H-Ras and gives rise to aberrant function in this oncogenic variant, which may be sufficient to interfere with the intrinsic regulation of downstream signaling.

The identification of specific conformations associated with distinct stable and semi-stable structural states in WT H-Ras and variants supports wet-laboratory efforts on selectively interfering with misfunctions in oncogenic variants [51]. Findings on the two variants studied here are important in understanding how mutations in Ras affect function and can be further applied to predict the effect of mutations that remain unclassified. Ras mutations vary in their oncogenicity, and the reason is not understood. Sampling variant-preferred conformational states may help in elucidating this challenging goal and is the subject of future studies in our labs. There are differences among Ras isoforms, as well, and future studies can focus on isoforms other than H-Ras.

We believe that the results presented in this paper have both confirmed experimental and computational knowledge on H-Ras, as well as advanced knowledge through novel findings. For the first time, energy landscapes have now been reported for the WT and two oncogenic variants of H-Ras. In addition, novel functional conformations have been reported. These novel findings are crucial to advance our understanding of H-Ras and facilitate other structure-driven studies in the wet or dry laboratories. For this reason, in addition to the implementation of SIfTER, which is available at http://www.cs.gmu.edu/~ashehu/?q=OurTools, all the data obtained and reported in this paper are available to researchers upon demand.

From a methodological point of view, the use of PCA in SIfTER is an effective means to reduce the search space and focus computational resources on structural fluctuations that have been captured in the wet laboratory. However, it also presents challenges that may limit its application. Sufficient experimental structures need to be deposited so dimensionality reduction techniques can be employed. It is also hard to draw general rules of thumb on how many structures and other considerations for application of PCA and credibility of the motions captured by its PCs. Investigation of these needs to be conducted on a case by case basis. For instance, an analysis along the lines of what we detail in the Supporting Information can be conducted to identify and exclude outlier structures whose inclusion would bias the PCA-revealed modes of motion away from those demonstrated to have functional implications in the wet laboratory. In addition, other techniques can be employed to extract concerted motions from one structure at a time.

It is worth noting that non-linear dimensionality techniques may possibly reveal even lower-dimensional search spaces than PCA, which is a linear dimensionality reduction technique, but they must allow conformational search algorithms direct sampling in the reduced space, which PCA directly provides. However, the reduced search space obtained via PCA here is sequence-independent and therefore can be explored to search for stable and semi-stable structural states of a given protein, which makes SIfTER a general algorithm.

Direct integration of more physics-based energy functions may provide more accurate representations of the energy landscapes computed by SIfTER. However, at this moment, physics-based engines largely limit interactions via scripts; in particular, there is no side-chain packing functionality in AMBER as opposed to a simple-to-use interface in Rosetta that can be integrated in external codes. For these reasons, the analysis in this paper on AMBER landscapes is based on short post-processing of SIfTER-obtained functional conformations.

## Materials and Methods

SIfTER is a population-based, memetic, cellular evolutionary algorithm (EA), which refers to a specific class of stochastic optimization algorithms with high sampling capability. Its conformational search starts with an initial population of *P* individuals or samples. The population is evolved over a fixed number *N* of generations towards individuals of high fitness. As such, SIfTER is a population-based EA. The initial population is seeded with known crystallographic structures of WT and variant sequences of a protein under investigation. These structures are first reduced to their CA-trace, extracting only the coordinates of their CA atoms, and then threaded onto the sequence of interest for which SIfTER seeks to sample the energy landscape. A dimensionality reduction technique is employed to obtain projections of these traces in a lower-dimensional space. These projections seed the initial population.

The axes revealed by the dimensionality reduction technique also define the reduced search space from which SIfTER draws samples in the subsequent generations. An asexual reproduction operator is used for this purpose. The operator essentially perturbs a parent in a randomly drawn vector in the reduced search space, thus obtaining a new sample or offspring.

Since offspring are essentially points in some low-dimensional search space, their energetic quality cannot be directly determined. Each offspring needs to be mapped to a conformation, whose energy can then be measured through some energy function. Therefore, each offspring is lifted from the reduced search space to the all-atom conformation space of the sequence under investigation. This is achieved through a multiscale technique, which first recovers the CA trace for the offspring, then reconstructs the backbone over the CA trace, and finally packs side chains onto the reconstructed backbone. The latter makes use of an energetic minimization technique in order to map an offspring to a nearby conformation residing in a local minimum of the all-atom energy surface. This entire process, which essentially improves the energetic quality of an offspring and also allows estimating its fitness, is also known as a local improvement operator. The employment of a local improvement operator makes SIfTER a memetic EA.

The reproductive and improvement operators result in *N* offspring. Only *N* individuals out of the *N* parents and their *N* offspring can survive to serve as parents in the next generation. Survival is based on the fitness of an individual, which is measured here using the Rosetta *score12* (all-atom) value of the conformation corresponding to an individual. Lower energies are considered higher fitness. SIfTER is based on an overlapping evolutionary model, where offspring compete with parents for survival. A selection operator pitches offspring only against parents and not against one another.

To avoid premature convergence to a few local minima, a known issue with stochastic optimization and which we have observed during our design of SIfTER, a local/decentralized selection operator is employed. The operator improves the likelihood that structurally-diverse offspring will survive and be selected to seed the next generation. Competition among offspring and parents is limited. Offspring compete with structurally-similar parents. Similarity is efficiently determined in the reduced space. The employment of a local selection operator makes SIfTER a cellular EA.

Details and analysis on each of the algorithmic components in SIfTERnow follow.

### Data Collection and Preparation

On the specific application of SIfTER on H-Ras, the PDB is queried for any structures of H-Ras. Only crystallographic structures are considered in order to reduce biasing the dimensionality reduction technique with small structural fluctuations present in NMR ensembles. The WT sequence of 166 amino acids of the H-Ras catalytic domain is obtained from the UniProt [17]. This sequence is used as reference to define the maximum sequence length. Out of all collected structures, only those corresponding to variant sequences with no more than 3 mutations over the WT are retained. Any structures with missing internal fragments are excluded.

Specifically, 86 structures fitting these criteria are identified and collected (PDB ids are listed in S1 Table in the Supporting Information). 46 of these structures, which represent the state of the PDB for H-Ras by 2009, have been used previously by McCammon and colleagues to analyze the essential modes of motion in H-Ras [25]. We decide to only allow SIfTER to exploit these same 46 structures, leaving the other 40 added to the PDB after 2009 to validate several results obtained by SIfTER (PDB ids are listed in S1 Table in the Supporting Information).

Our premise is to treat these 46 structures as known representatives of stable or semi-stable structural states in any sequence of H-Ras, whether WT or variant. SIfTER does so by threading CA traces of these structures onto a sequence of interest. The traces are subjected to the dimensionality reduction technique described next to define the reduced search space. They are also employed to seed the initial population.

### Defining the Reduced Search Space for SIfTER

Like McCammon and colleagues [25], we also employ PCA [52] as our dimensionality reduction technique. However, while McCammon and colleagues employed PCA mainly to visualize a collection of H-Ras structures on a two-dimensional map, SIfTER makes use of PCA to define its reduced search space for sampling novel conformations.

PCA finds orthogonal axes (Principal Components—PCs) in order of preserving variance. We subject PCA to the 46 CA traces in order to define the reduced search space. To ensure that the PCA results are not capturing rotational or translational differences but instead internal structural fluctuations, the CA traces are aligned to some reference trace (we use arbitrarily the first one) using the optimal superimposition process typically employed when identifying least root-mean-squared-deviation (lRMSD) between two structures [45]. Subsequent to the alignment, an average trace *AT* is computed and subtracted from all the traces. The resulting centered matrix *X* is subjected to the *dgesvd* routine in LAPACK [53] in order to obtain a singular value decomposition *X* = *U* ⋅ Σ ⋅ *V* ^*T*^. The new axes or PCs are the rows of the *U* matrix, and the singular values, which are the square roots of the eigenvalues corresponding to the PCs, are the diagonal entries of the Σ matrix. The PCs are ordered from largest to smallest corresponding eigenvalue; an eigenvalue measures the variance captured by the corresponding PC if the data (traces aligned and centered) are projected onto it.

An aligned CA trace (*CT*), even if not included in the PCA, can be readily projected onto the space of extracted PCs. Its projection *RS* can be obtained using the equation *RS* = (*CT* − *AT*) ⋅ *U*. Conversely, an aligned CA trace *CT* can be recovered from a projection *RS* by the following equation *CT* = *RS* ⋅ *U* ^*T*^ + *AT*. These two equations are important for SIfTER to have the sample drawing and the multiscale procedure interface with each-other seamlessly. When a sample/offspring is generated, its CA trace can be recovered via the second equation. When the multiscale procedure is applied on a CA trace and an all-atom conformation is obtained, its projection back onto the reduced search space can be obtained via the first equation. Projecting all-atom conformations back onto the reduced space is necessary, as the multiscale procedure may slightly modify the CA trace in order to accommodate side chains for an overall lower all-atom energy.

### Determination of Effectiveness of PCA for H-Ras

Analysis of eigenvalues allows determining whether PCA is effective, which is not guaranteed if the data lie on a non-linear space. As originally demonstrated by McCammon and colleagues [25] and also by us here, more than 90% of the variance can be captured with no more than 10 PCs; that is, if the traces are instead represented by their projections on 10 axes. The top two PCs capture more than 50% of the cumulative variance (as related in Fig 1 in the Results section). The detailed analysis of the motions captured by the PCs in the Results section allows concluding that PCA is effective for H-Ras, and that the PCs can be employed as axes of a reduced search space to search for novel functional conformations of a given sequence of H-Ras.

### Determination of Dimensionality of Reduced Search Space

The fact that PCA is effective means that SIfTER can operate not in the full space of 166 × 3 dimensions but instead on a lower-dimensional space of *d* PCs of corresponding highest eigenvalues. This effectively allows SIfTER to represent an individual in its search by only *d* collective coordinates, but determining an effective value for *d* is critical. In S1 Text in the Supporting Information we outline a procedure for doing so by employing the additional 40 structures/traces not subjected to the PCA. Analysis of data obtained from the procedure (shown in S2 Fig in the Supporting Information) suggests *d* = 10 for the dimensionality of the search space. SIfTER directly generates 10-dimensional samples in the space of the top ten PCs revealed by the PCA; that is, each individual is represented by only 10 variables that are projections on the top 10 PCs.

### Asexual Reproduction Operator over Reduced Representation in SIfTER

The reproductive operator perturbs each parent, one at a time, in a randomly-drawn vector in the *d*-dimensional search space to obtain an offspring for each parent. A maximum step size *s*_max_ (set to 1 here) is first defined. For each of the *d* PC coordinates of the parent, a step size *s*_*i*_ is sampled uniformly in [−*s*_max_, +*s*_max_] and then scaled according to the ratio of variance captured by the corresponding *PC*_*i*_ such that $s_{i,scaled}=s_{i}\cdot \frac{Var(PC_{i})}{Var(PC_{1})}$. Since the PC dimensions are ordered according to the variance they capture (highest to lowest), scaling the step size in each dimension in this way ensures that larger perturbations will be carried out along the PCs/dimensions that capture more of the variance. This essentially preserves the shape of the search space as suggested by the crystallographic structures. After the step size for each dimension is determined in this way, the corresponding coordinate *PC*_*i*,*offspring*_ for the offspring is obtained by *PC*_*i*,*offspring*_ = *PC*_*i*,*parent*_+*s*_*i*,*scaled*_.

### Local Improvement Operator in SIfTER

Each offspring obtained by the reproductive operator is subjected to a local improvement operator. The process begins with recovering the CA trace corresponding to the *d*-dimensional representation of an offspring, as detailed above. A backbone is then reconstructed from the CA trace using BBQ [54], which is one of the top backbone reconstruction protocols. Our decision to employ BBQ over other similar protocols is due to the reported ability of BBQ to faithfully restore backbones [54]. Once the backbone is built from a CA trace, side chains are then packed onto the reconstructed backbone via the Rosetta *relax* protocol [55]. The protocol is employed to obtain an all-atom conformation corresponding to the offspring drawn by the reproductive operator in the reduced search space. In addition to adding side chains, the *relax* protocol conducts a Monte-Carlo based energetic minimization of the all-atom conformation to obtain an all-atom conformation representing a local minimum in the all-atom energy landscape. While there are currently many side-chain packing protocols, the one in the Rosetta software package employs a sophisticated all-atom energy function as opposed to simple functions focusing mainly on Lennard-Jones and electrostatic interactions. In addition, the protocol is efficient and implemented in an object-oriented programming language, which allows efficient interfacing with our implementation of SIfTER and maintaining the computational demands of the algorithm low. Analysis on the effectiveness of the local improvement operator is provided in Supporting Information in S3 and S4 Figs.

The ability to integrate Rosetta functionality in SIfTER is one of the main reasons for choosing Rosetta as opposed to physics-based simulation platforms to evaluate and energetically-refine conformations. The latter require scripting, which results in computationally impractical time demands for an algorithm that essentially generates *N* × *P* all-atom conformations. It is also important to note that there is currently no side-chain packing functionality in AMBER; that is, to pack side chains onto backbone structures, one needs to rely on other packages. This is a central reason why we use Rosetta in this paper. However, we do address the generality of the obtained Rosetta *score12* landscapes by further subjecting all generated conformations to short energetic minimizations in AMBER. The minimization protocol uses the Amber *ff12SB* force field and *sander* to conduct 500 steps of steepest descent followed by 500 steps of conjugate gradient descent (*maxcyc = 1000, ncyc = 500*). Nonbonded interactions beyond 10Å are cutoff (*cutoff = 10*). The generalized Born solvation model is used (*igb = 1*). All our conclusions regarding changes that mutations introduce to the H-Ras WT energy landscape are made by studying common features between the Rosetta *score12* and the AMBER *ff12SB* landscapes.

### Local Selection Operator in SIfTER

Due to the employment of the Rosetta *relax* protocol in the local improvement operator, the fitness value that the selection operator in SIfTER uses to evaluate and compare individuals is the all-atom Rosetta *score12* energy. Given two individuals under comparison, the one with the highest fitness (lowest *score12* value) survives. Instead of a global or central selection operator, SIfTER employs a local or decentralized one. A global selection operator combines all *N* offspring and *N* parents in a generation prior to determining which *N* should survive based on fitness. The danger with such an operator is that offspring have a hard time competing with parents. Therefore, the entire algorithm risks being taken over very quickly by a few currently fittest individuals, essentially prematurely converging to a few local minima.

Since the goal in SIfTER is high sampling capability so as not to miss important functional conformations), a local selection operator is employed to improve the likelihood that offspring survive. This is accomplished through what is known as a *crowding model* [56], where essentially offspring compete with a limited subset of parents. The idea is to have an offspring compete mainly with structurally-similar parents. Structural similarity is determined quickly and coarsely over a 2-dimensional representation of individuals; essentially, only the first two coordinates (top two PCs) are used, so that a simple 2-dimensional grid can be imposed over parents and offspring. Individuals in the same or nearby cells are considered structurally-similar. If there are no parents nearby, an offspring competes with all parents. The concept of a neighborhood is illustrated in the Supporting Information in the top panel of S6 Fig.

A detailed analysis is conducted to determine an effective neighborhood size *C*, also detailed in the Supporting Information. The analysis suggests employing a value of 25, which is what is used to obtain the data reported and analyzed in this paper (the analysis provided in the Supporting Information in the bottom panel of S6 Fig also shows that convergence is reached by generation 50, suggesting that any number of generations no smaller than this value is sufficient to allow SIfTER to explore the breadth of the conformation space.)

### Initial Population

The 46 crystallographic structures used to define its *d*-dimensional search space seed the initial population. Their projections are the first set of individuals added to the initial population. To associate fitness values with these individuals, each of them is subjected to the local improvement operator. Moreover, SIfTER uses a much larger population size *P* = 500. The size of the population is an important decision, as a small population risks premature convergence, whereas a larger one increases the computational demands of an EA. Typically, population sizes in the hundreds are currently advised for application of EAs on medium-size proteins (cf. to Review in Ref. [31]). Analysis of applications of SIfTER with smaller population sizes (data not shown) have led us to *P* = 500 as a compromise between obtaining a broad view of the conformation space while controlling the computational demands of the algorithm to a few days on one CPU.

To increase the size of the initial population from 46 employed crystallographic structures to 500, more individuals need to be generated. *P* is continually doubled by subjecting all current individuals to the reproductive and local improvement operator before being added back into the population. This continues until doubling again would cause the population to exceed *P* = 500. The population is then filled to the desire size by continuing to randomly select an individual to generate another offspring, which is added to the population.

### Implementation Details

The algorithm is implemented in C/C++ and run on a 16 core red hat linux box with 3.2GhZ HT Xeon CPU and 8GB RAM. Population size *P* is set to 500, and SIfTER is run for *N* = 100 generations. The analysis summarized in the Supporting Information (bottom panel of S6 Fig) indicates that this number of generations is sufficient to allow SIfTER to converge; indeed, convergence is observed around generation 50. The reproductive operator uses a maximum step size of 1. The local selection operator uses neighborhood *C*25, and cell widths of 1. Total run time for application of SIfTER on a given Ras sequence is approximately 72 hours on 16 CPUs (16 processes are used to alleviate the computation burden of the Rosetta *relax* protocol employed when improving offspring). Finally, it is worth noting that the results shown in this paper are not exploiting particular runs of SIfTER. Instead, the algorithm is run many times, and comparison of energy landscapes and convergence across the different runs (data shown in Supporting Information in S7 Fig) allow us to conclude that the results presented here are representative of the capabilities of the algorithm and reproducible.

## Supporting Information

S1 Text Supporting Information Text. The text first lists all abbreviations used in the manuscript and then provides further details on preparation of data subjected to PCA, determination of the dimensionality of the search space explored by SIfTER, effectiveness of the local improvement operator, determination of the neighborhood parameter in the local selection operator, and analysis on the robustness of SIfTER, added value of populations, and energetic variance.
(PDF)

S1 Table List of PDB Ids of Crystallographic Structures. The list of PDB ids corresponding to crystallographic structures extracted from the PDB for H-Ras (WT and variants) is shown. Structures used by the PCA are labeled either GTP or GDP. Structures withheld from the PCA but used for validation are labeled Validation.
(PDF)

S2 Table Structures Deemed Outliers. PDB ids of 5 crystallographic structures deemed outliers are listed here, together with information on the papers introducing them to show the two labs contributing them to the PDB.
(PDF)

S1 Fig Visualization of Outlier Structures. The 5 crystallographic structures deemed outliers are shown here in red (PDB ids 4EFM, 4EFL, 4EFN, 3KKN) and orange (PDB id 1BKD), superimposed over a representative structure (drawn in green). The SI and SII regions are denoted.
(TIFF)

S2 Fig RMSDs between Original and *d*-reconstructed CA traces. Distributions of RMSDs between original, *CT*, and *d*-reconstructed CA traces, *CT*_*d*_, are shown here for *d* ∈ {5, 7, 10}. The analysis is over all 86 crystallographic structures collected for H-Ras, including the 40 structures withheld from PCA.
(TIFF)

S3 Fig RMSD Deviations from Multiscale Procedure and Relaxation. What is shown here is the distortion in backbone RMSD resulting from projecting a crystallographic structure into the PC space, rebuilding the CA trace using 10 PCs, rebuilding the backbone using BBQ, and finally adding back the side chains and doing a short minimization with the Rosetta *relax* protocol.
(TIFF)

S4 Fig Deviations from Multiscale Procedure and Relaxation in Reduced Space. Magnitude and direction along which the Rosetta energy function wants to move crystallographic structures in the *score12* all-atom landscape are shown here. The process is repeated for each sequence of H-Ras considered here, with the WT shown in the top panel, G12V in the middle panel, and Q61L in the bottom panel.
(TIFF)

S5 Fig Deviation from Amber Minimization. Change due to Amber minimization protocol is shown here for all functional conformations obtained by SIfTER for WT H-Ras in terms of CA RMSD.
(TIFF)

S6 Fig Role of Neighborhood Size on Population Diversity. Top panel: The C1 (left), C9 (middle), and C25 (right) neighborhoods are illustrated here. The cell populated by the offspring is drawn in red. Cells in green are additional neighboring cells considered by the local selection operator when increasing the *C* parameter. Bottom panel: The structural diversity of the population in each generation is tracked across 100 generations. This is done for five settings of *C* in the local selection operator: *C*1, *C*9, *C*25, *C*49, and *C*∞. The latter corresponds to global selection.
(TIFF)

S7 Fig Comparison of Landscapes and Energies Obtained from Different Runs of SIfTER. Top panel: Three landscapes are shown here for H-Ras WT obtained from three different runs of SIfTER Bottom panel: Distributions of energies obtained on the H-Ras WT from three different runs of SIfTER are superimposed over one another.
(TIFF)

S8 Fig Additional Populations. The energy landscape associated with functional conformations generated by SIfTER for WT H-Ras is shown here, together with the conformations of the initial population. The latter are color-coded according to their energetic difference from the lowest-energy conformation among the functional conformations. It can be seen that additional populations in SIfTER are needed to fill in regions of the conformation space (and associated energy landscape) not covered by either the crystallographic structures or the additional ones obtained by perturbing them in the initial population.
(TIFF)

S9 Fig Energy Landscapes Along PC3. Projections are shown along PC3, as well, for each of the three sequences. The color-coding is as described in the manuscript. The states are labeled to the extent that they are visible along PC3.
(TIFF)

S10 Fig Energetic Variance Analysis. The variance of the energy values behind each cell in the grid imposed over PC1 and PC2 for visualization of the energy landscapes is shown here as follows: instead of color-coding each cell according to the median value over energies of conformations mapping to it, the variance is used instead. This is done for each of the three sequences.
(TIFF)

## References

[1] Hamosh A, Scott AF, Amberger JS, Bocchini CA, McKusick VA. Online Mendelian Inheritance in Man (OMIM), a knowledgebase of human genes and genetic disorders. Nucleic Acids Res. 2005;1(33):D514–D517. 10.1093/nar/gki033 PMC539987 15608251

[2] Stenson PD, Mort M, Ball EV, Shaw K, Phillips A, Cooper DN. The Human Gene Mutation Database: building a comprehensive mutation repository for clinical and molecular genetics, diagnostic testing and personalized genomic medicine. Hum Genet. 2014;133(1):1–9. 10.1007/s00439-013-1358-4 24077912 PMC3898141

[3] Karplus M, Kuriyan J. Molecular dynamics and protein function. Proc Natl Acad Sci USA. 2005;102(19):6679–6685. 10.1073/pnas.0408930102 15870208 PMC1100762

[4] Amaro RE, Bansai M. Editorial overview: Theory and simulation: Tools for solving the insolvable. Curr Opinion Struct Biol. 2014;25:4–5. 10.1016/j.sbi.2014.04.004 24835772

[5] Karnoub AE, Weinberg RA. Ras oncogenes: split personalities. Nat Rev Mol Cell Biol. 2008 7;9(7):517–531. 10.1038/nrm2438 18568040 PMC3915522

[6] Berman HM, Henrick K, Nakamura H. Announcing the worldwide Protein Data Bank. Nat Struct Biol. 2003;10(12):980–980. 10.1038/nsb1203-980 14634627

[7] Grant BJ, Gorfe AA, McCammon JA. Ras Conformational Switching: Simulating Nucleotide-Dependent Conformational Transitions with Accelerated Molecular Dynamics. PLoS Comput Biol. 2009;5(3):e1000325 10.1371/journal.pcbi.1000325 19300489 PMC2651530

[8] Vetter IR, Wittinghofer A. The guanine nucleotide-binding switch in three dimensions. Science. 2001 11;294(5545):1299–1304. 10.1126/science.1062023 11701921

[9] Nassar N, Horn G, Herrmann C, Scherer A, McCormick F, Wittinghofer A. The 2.2 A crystal structure of the Ras-binding domain of the serine/threonine kinase c-Raf1 in complex with Rap1A and a GTP analogue. Nature. 1995 6;375(6532):554–560. 10.1038/375554a0 7791872

[10] Ford B, Skowronek K, Boykevisch S, Bar-Sagi D, Nassar N. Structure of the G60A mutant of Ras: implications for the dominant negative effect. J Biol Chem. 2005 7;280(27):25697–25705. 10.1074/jbc.M502240200 15878843

[11] Hall BE, Bar-Sagi D, Nassar N. The structural basis for the transition from Ras-GTP to Ras-GDP. Proc Natl Acad Sci USA. 2002 9;99(19):12138–12142. 10.1073/pnas.192453199 12213964 PMC129411

[12] Ford B, Hornak V, Kleinman H, Nassar N. Structure of a transient intermediate for GTP hydrolysis by ras. Structure. 2006 3;14(3):427–436. 10.1016/j.str.2005.12.010 16531227

[13] Nassar N, Cancelas J, Zheng J, Williams DA, Zheng Y. Structure-function based design of small molecule inhibitors targeting Rho family GTPases. Curr Top Med Chem. 2006;6(11):1109–1116. 10.2174/156802606777812095 16842149

[14] Rohrer M, Prisner TF, Brugmann O, Kass H, Spoerner M, Wittinghofer A, et al Structure of the metal-water complex in Ras x GDP studied by high-field EPR spectroscopy and 31P NMR spectroscopy. Biochemistry. 2001 2;40(7):1884–1889. 10.1021/bi002164y 11329253

[15] Spoerner M, Herrmann C, Vetter IR, Kalbitzer HR, Wittinghofer A. Dynamic properties of the Ras switch I region and its importance for binding to effectors. Proc Natl Acad Sci USA. 2001 4;98(9):4944–4949. 10.1073/pnas.081441398 11320243 PMC33143

[16] Barbacid M. ras genes. Annu Rev Biochem. 1987;56:779–827. 10.1146/annurev.bi.56.070187.004023 3304147

[17] Magrane M, the UniProt consortium. UniProt Knowledgebase: a hub of integrated protein data. Database. 2011;2011(bar009):1–13. 10.1093/database/bar009 PMC3070428 21447597

[18] Rajalingam K, Schreck R, Rapp UR, Albert S. Ras oncogenes and their downstream targets. Biochim Biophys Acta. 2007 8;1773(8):1177–1195. 10.1016/j.bbamcr.2007.01.012 17428555

[19] Buhrman G, Wink G, Mattos C. Transformation efficiency of RasQ61 mutants linked to structural features of the switch regions in the presence of Raf. Structure. 2007 12;15(12):1618–1629. 10.1016/j.str.2007.10.011 18073111 PMC2273997

[20] Buhrman G, Holzapfel G, Fetics S, Mattos C. Allosteric modulation of Ras positions Q61 for a direct role in catalysis. Proc Natl Acad Sci USA. 2010 3;107(11):4931–4936. 10.1073/pnas.0912226107 20194776 PMC2841912

[21] O’Connor C, Kovrigin EL. Global conformational dynamics in ras. Biochemistry. 2008 9;47(39):10244–10246. 10.1021/bi801076c 18771285

[22] Baussand J, Kleinjung J. Specific Conformational States of Ras GTPase upon Effector Binding. J Chem Theory Comput. 2013 1;9(1):738–749. 10.1021/ct3007265 23316125 PMC3541755

[23] Foley CK, Pedersen LG, Charifson PS, Darden TA, Wittinghofer A, Pai EF, et al Simulation of the solution structure of the H-ras p21-GTP complex. Biochemistry. 1992 6;31(21):4951–4959. 10.1021/bi00136a005 1599919

[24] Diaz JF, Wroblowski B, Engelborghs Y. Molecular dynamics simulation of the solution structures of Ha-ras-p21 GDP and GTP complexes: flexibility, possible hinges, and levers of the conformational transition. Biochemistry. 1995 9;34(37):12038–12047. 10.1021/bi00037a047 7547942

[25] Gorfe AA, Grant BJ, McCammon JA. Mapping the nucleotide and isoform-dependent structural and dynamical features of Ras proteins. Structure. 2008 6;16(6):885–896. 10.1016/j.str.2008.03.009 18547521 PMC2519881

[26] Ma J, Karplus M. Molecular switch in signal transduction: reaction paths of the conformational changes in ras p21. Proc Natl Acad Sci USA. 1997 10;94(22):11905–11910. 10.1073/pnas.94.22.11905 9342335 PMC23651

[27] Diaz JF, Wroblowski B, Schlitter J, Engelborghs Y. Calculation of pathways for the conformational transition between the GTP- and GDP-bound states of the Ha-ras-p21 protein: calculations with explicit solvent simulations and comparison with calculations in vacuum. Proteins. 1997 7;28(3):434–451. 10.1002/(SICI)1097-0134(199707)28:3%3C434::AID-PROT12%3E3.3.CO;2-T 9223188

[28] Hamelberg D, Mongan J, McCammon JA. Accelerated molecular dynamics: a promising and efficient simulation method for biomolecules. J Chem Phys. 2004 6;120(24):11919–11929. 10.1063/1.1755656 15268227

[29] Lukman S, Grant BJ, Gorfe AA, Grant GH, McCammon JA. The distinct conformational dynamics of K-Ras and H-Ras A59G. PLoS Comput Biol. 2010;6(9). 10.1371/journal.pcbi.1000922 20838576 PMC2936511

[30] Grant BJ, Lukman S, Hocker HJ, Sayyah J, Brown JH, McCammon JA, et al Novel allosteric sites on Ras for lead generation. PLoS ONE. 2011;6(10):e25711 10.1371/journal.pone.0025711 22046245 PMC3201956

[31] Shehu A. Probabilistic Search and Optimization for Protein Energy Landscapes In: Aluru S, Singh A, editors. Handbook of Computational Molecular Biology. Chapman & Hall/CRC Computer & Information Science Series; 2013.

[32] Noe F, Ille F, Smith JC, Fischer S. Automated computation of low-energy pathways for complex rearrangements in proteins: application to the conformational switch of Ras p21. Proteins. 2005 5;59(3):534–544. 10.1002/prot.20422 15778967

[33] Fischer S, Karplus M. Conjugate Peak Refinement: an algorithm for finding reaction paths and accurate transition states in systems with many degrees of freedom. Chem Phys Lett. 1992;194:252–261. 10.1016/0009-2614(92)85543-J

[34] de Groot BL, van Aalten DM, Scheek RM, Amadei A, Vriend G, Berendsen HJ. Prediction of protein conformational freedom from distance constraints. Proteins. 1997;29(2):240–251. 10.1002/(SICI)1097-0134(199710)29:2%3C240::AID-PROT11%3E3.0.CO;2-O 9329088

[35] Wells SA. Geometric simulation of flexible motion in proteins. Methods Mol Biol. 2014;1084:173–192. 10.1007/978-1-62703-658-0_10 24061922

[36] Wells SA, Menor S, Hespenheide B, Thorpe MF. Constrained geometric simulation of diffusive motion in proteins. Phys Biol. 2005;4(4):S127–S136. 10.1088/1478-3975/2/4/S07 16280618

[37] Shehu A, Clementi C, Kavraki LE. Modeling Protein Conformational Ensembles: From Missing Loops to Equilibrium Fluctuations. Proteins: Struct Funct Bioinf. 2006;65(1):164–179. 10.1002/prot.21060 16917941

[38] Shehu A, Clementi C, Kavraki LE. Sampling Conformation Space to Model Equilibrium Fluctuations in Proteins. Algorithmica. 2007;48(4):303–327. 10.1007/s00453-007-0178-0

[39] Shehu A, Kavraki LE, Clementi C. On the Characterization of Protein Native State Ensembles. Biophys J. 2007;92(5):1503–1511. 10.1529/biophysj.106.094409 17158570 PMC1796840

[40] Boehr DD, Nussinov R, Wright PE. The role of dynamic conformational ensembles in biomolecular recognition. Nat Chem Biol. 2009 11;5(11):789–796. 10.1038/nchembio.232 19841628 PMC2916928

[41] Tsai CJ, Ma B, Nussinov R. Folding and binding cascades: shifts in energy landscapes. Proc Natl Acad Sci USA. 1999 8;96(18):9970–9972. 10.1073/pnas.96.18.9970 10468538 PMC33715

[42] Tsai CJ, Kumar S, Ma B, Nussinov R. Folding funnels, binding funnels, and protein function. Protein Sci. 1999 6;8(6):1181–1190. 10.1110/ps.8.6.1181 10386868 PMC2144348

[43] Ma B, Kumar S, Tsai CJ, Nussinov R. Folding funnels and binding mechanisms. Protein Eng. 1999 9;12(9):713–720. 10.1093/protein/12.9.713 10506280

[44] Schrödinger, LLC. The PyMOL Molecular Graphics System, Version 1.3r1; 2010.

[45] McLachlan AD. A mathematical procedure for superimposing atomic coordinates of proteins. Acta Crystallogr A. 1972;26(6):656–657. 10.1107/S0567739472001627

[46] Tong LA, de Vos AM, Milburn MV, Kim SH. Crystal structures at 2.2 A resolution of the catalytic domains of normal ras protein and an oncogenic mutant complexed with GDP. J Mol Biol. 1991 2;217(3):503–516. 10.1016/0022-2836(91)90753-S 1899707

[47] Hall BE, Bar-Sagi D, Nassar N. The structural basis for the transition from Ras-GTP to Ras-GDP. Proc Natl Acad Sci USA. 2002 9;99(19):12138–12142. 10.1073/pnas.192453199 12213964 PMC129411

[48] Al-Mulla F, Milner-White EJ, Going JJ, Birnie GD. Structural differences between valine-12 and aspartate-12 Ras proteins may modify carcinoma aggression. J Pathol. 1999;187(4):433–438. 10.1002/(SICI)1096-9896(199903)187:4%3C433::AID-PATH273%3E3.0.CO;2-E 10398103

[49] Krengel U, Schlichting I, Scherer A, Schumann R, Frech M, John J, et al Three-dimensional structures of H-ras p21 mutants: molecular basis for their inability to function as signal switch molecules. Cell. 1990;62(3):539–548. 10.1016/0092-8674(90)90018-A 2199064

[50] Fetics SK, Guterres H, Kearney BM, Buhrman G, Ma B, Nussinov R, et al Allosteric Effects of the Oncogenic RasQ61L Mutant on Raf-RBD. Structure. 2015;23(3):505–516. 10.1016/j.str.2014.12.017 25684575 PMC7755167

[51] Marcus K, Mattos C. Direct Attack on RAS: Intramolecular Communication and Mutation-Specific Effects. Clin Cancer Res. 2015;21:1810 10.1158/1078-0432.CCR-14-2148 25878362

[52] Luenberger DG. Introduction to Linear and Nonlinear Programming. Addison-Wesley; 1973.

[53] Anderson E, Bai Z, Dongarra J, Greenbaum A, McKenney A, Du Croz J, et al. LAPACK: A Portable Linear Algebra Library for High-performance Computers. In: Proceedings of the 1990 ACM/IEEE Conference on Supercomputing. Supercomputing ‘90. Los Alamitos, CA, USA: IEEE Computer Society Press; 1990. p. 2–11. Available from: http://dl.acm.org/citation.cfm?id=110382.110385.

[54] Gront D, Kmiecik S, Kolinski A. Backbone building from quadrilaterals: a fast and accurate algorithm for protein backbone reconstruction from alpha carbon coordinates. J Comput Chem. 2007;28(29):1593–1597. 10.1002/jcc.20624 17342707

[55] Kaufmann KW, Lemmon GH, DeLuca SL, Sheehan JH, Meiler J. Practically Useful: What the Rosetta Protein Modeling Suite Can Do for You. Biochemistry. 2010;49(14):2987–2998. 10.1021/bi902153g 20235548 PMC2850155

[56] Mengshoel OJ, Goldberg DE. The crowding approach to niching in genetic algorithms. Evol Comput. 2008;16(3):315–354. 10.1162/evco.2008.16.3.315 18811245

