# Supplementary material for: Mapping the Conformation Space of Wildtype and Mutant H-Ras with a Memetic, Cellular, and Multiscale Evolutionary Algorithm
Source: PLoS Comput Biol. 2015 Sep 1;11(9):e1004470. doi: 10.1371/journal.pcbi.1004470 (PMC4556523; doi:10.1371/journal.pcbi.1004470)
Supplement: S1 Text — The text first lists all abbreviations used in the manuscript and then provides further details on preparation of data subjected to PCA, determination of the dimensionality of the search space explored by SIfTER, effectiveness of the local improvement operator, determination of the neighborhood parameter in the local selection operator, and analysis on the robustness of SIfTER, added value of populations, and energetic variance. (PDF) [file pcbi.1004470.s001.pdf]

Supporting Information

S1 Text

Abbreviations WT, **W**ild**t**ype; PDB, **P**rotein **D**ata **B**ank; SIfTER, **S**tructure **I**nitiated Search **f**or **T**ransient **E**nergy **R**egions; BBQ, **B**ackbone **B**uilding from **Q**uadrilaterals

Data Preparation The list of PDB ids corresponding to the 86crystallographic structures extracted from the PDB for H-Ras (WT and variants) is shown in S1 Table. Structures used by the PCA are labeled either GTP or GDP. These structures are those in the PDB prior to 2009, employed and analyzed via PCA originally in [25] and shown to produce PCs that captured the structural motions between the On and Off structural states in the catalytic domain of H-Ras. Our analysis in the manuscript shows that the structural information contained in these structures is sufficient to allow the algorithm to largely reproduce the structures added to the PDB afterwards, with the exception of 5 structures. Instead of adding these structures for analysis via PCA, we decide to exclude them, as our structural analysis indicates they are outliers. In particular, these are structures with PDB ids 4EFM, 4EFL, 4EFN, 3KKN, and 1BKD. These structures have all been added to the PDB after 2010, with the exception of the one with PDB id 1BKD, which has a deposit date of 1998. This structure, though in the PDB in 2009, was deliberately excluded from analysis by McCammon and colleagues in [25]. A reason is not provided in [25], but later similar work on H-Ras in [7] states that 1BKD has a strikingly open loop2-SI conformation not observed among other existing structures of H-Ras. This can be seen in S1 Fig, where we show this structure and the other 4 added to the PDB after 2010, which we have also deemed outliers.

These 5 structures are contributed from 2 labs, as shown in S2 Table. The structures have a large deviation on residues 26−37, which are part of the SI region. The structure with PDB id 1BKD, drawn in orange in S1 Fig, has more pronounced structural differences than the other 4 that are not consistent with motions attributed to the conformational switching in H-Ras (as observed among other GTP- and GDP-bound crystallographic structures). As such, these 5 structures can be considered outliers. We exclude them from PCA. Not only do these structures not agree with the known conformational switching, but if one were to include them, the structural change present in them is so large that it would be reflected in PC1 and overpower the structural change incurred by H-Ras for its conformational switch between the On and Off states.

Determination of Dimensionality of Reduced Search Space for SIfTER Let's consider a specific value $1 \leq d \leq 166 * 3$. The accumulation of variance plot in Fig. 1 in the main text suggests a maximal value can be $d = 10$ (with 10 PCs, one captures more than 90% of the variance of the original data). Two other values that can be considered are $d = 5$ (with 5 PCs one captures close

to 80% of the variance) and $d = 7$ (with 7 PCs one captures 85% of the variance). Given a value of d, each trace CT can be projected onto the d PCs, as described above. The projection RS_d can then be mapped back to a trace CT_d, also as described above. Note that CT and CT_d will only be identical if $d = 166 \times 3$. The distortion that considering a smaller value of d introduces can be directly measured through the RMSD between CT and CT_d. This can be done for each of the 86 structures (including those not directly used by PCA to obtain the PCs), and the distribution of resulting RMSD values can be analyzed to estimate the amount of distortion.

S2 Fig shows such distributions for $d \in \{5, 7, 10\}$. A bimodal distribution is observed for $d = 5$ and $d = 7$. This is a result of the fact that the original 46 traces used to obtain the PCs are, as expected, reconstructed more accurately than the second set of 40 traces withheld from the PCA. About 5 of these "withheld" traces are reconstructed with a 1.9 to 3Å difference, which is due to a large structural change in a single loop of the catalytic domain of H-Ras not observed among the rest of the H-Ras crystallographic structures. A close to unimodal distribution is observed for $d = 10$, which is the reason why we employ $d = 10$ as the dimensionality of the search space over which SIfTER draws samples; thus, representing each individual by only 10 variables that are projections on the top 10 PCs.

Effectiveness of the Local Improvement Operator The *relax* protocol allows constraining motions of the backbone, so the search is mainly conducted over side-chain configurations. We employ such an option here, as we want to obtain a conformation whose location in the reduced space remains close to the corresponding offspring. Given that all side-chain packing protocols that employ sophisticated energy functions are stochastic as opposed to exact, the relationship between a reduced representation and the all-atom representation is not one to one. However, constraining motions of the backbone allows establishing a correspondence between a point drawn in the reduced space by SIfTER and a nearby local minimum in the all-atom energy surface.

By constraining motions of the backbone, the deviations between the location of the offspring and the location of the corresponding all-atom conformation obtained for it in this way can also be rigorously measured. We do so by investigating the ability to rebuild a given crystallographic structure for H-Ras from its reduced d-dimensional representation (with d valued at 10, as described above). Each of the 86 crystallographic structures (including the 40 not subjected to PCA) is projected onto $d = 10$ dimensions. The local improvement operator, as described, is subjected to each projection to recover an all-atom conformation for each projection. The original crystallographic structure is then compared in terms of RMSD to the all-atom conformation obtained by the local improvement operator. Only backbone RMSD can be measured, as some of the original crystallographic structures have missing atoms in various side chains. The distribution of RMSDs is plotted in S3 Fig, which shows that all 86 crystallographic structures are reconstructed within 1Å, with the exception

of the 5 outlier structures noted above. A deviation of this size is rather small for a protein of 166 amino acids. It is also expected, as noted by Baker and colleagues [1].

The analysis provided in S3 Fig essentially suggests that the location that the multiscale procedure assigns to a conformation in the all-atom energy landscape may be within 1Å or less of its true location (in terms of backbone RMSD). Controlling this deviation is important in order to be able to make credible comparisons regarding locations of basins, barriers, and other features of energy landscapes mapped by SIfTER for different H-Ras sequences.

Deviations in Structure Reconstruction due to Multiscale Procedure and Relaxation in Local Improvement Operator The extent of the deviation from the multiscale procedure used to build all-atom models of the crystallographic structures is shown for each of the three H-RAS sequences in S4 Fig. For a particular sequence, crystallographic structures deposited for other sequences are minimally corrected to remove incompatible side-chain atoms. The Rosetta *relax* protocol is then applied 500 times to each resulting structure to obtain the extent of deviations; that is, the magnitude and direction along which the Rosetta *score12* energy function wants to shift the positions of true local minima corresponding to the crystallographic structures. The magnitude of the deviation is drawn through ellipsoids, each centered at the projection of the CA traces corresponding to the crystallographic structures. The radii of each ellipsoid are the standard deviations along each of the axes, PC1 and PC2. The color-coding of the ellipsoids follows the energy scale shown on the right, where the energy of all 500 models obtained per crystal structure is averaged to associate an average energy score to each ellipsoid. The arrows drawn for each ellipsoid show the direction of the movement in the PC1-PC2 map from the *relax* protocol.

S4 Fig demonstrates that there are crystallographic structures which Rosetta wants to shift to different locations in the *score12* landscape. However, the majority of structures are kept nearby, which suggests that the Rosetta *score12* energy landscape is close to the true one for the H-RAS sequences considered in this study.

Deviations from Amber Minimization S5 Fig shows the structural changes introduced by the Amber minimization protocol used here. The CA RMSDs between SIfTER-generated functional conformations for WT H-Ras before and after the minimization protocol are shown on the left and right panels, respectively, of S5 Fig. Very slight structural changes around a mean of 0.22Å and not higher than 0.40Å are observed. This is not surprising, particularly since these conformations are already local minima in the Rosetta *score12* energy landscape.

Determination of Neighborhood Parameter Value S6 Fig illustrates various neighborhoods (top panel) that can be defined, using a configurable

neighborhood size parameter C. When $C = 1$, only parents in the immediate cell where the offspring maps in the 2-dimensional grid compete with the offspring. With larger C, the pool of parents competing with the offspring increases. To determine the neighborhood size, SIfTER is applied to the WT sequence five independent times, using neighborhood sizes of $C1$, $C9$, $C25$, $C49$, and $C\infty$ (in $c\infty$, the local selection operator becomes a global selection operator). The structural diversity of a population is tracked over the generations for each of these 5 settings and plotted in the bottom panel of S6 Fig. Structural diversity of a population is measured as the average CA RMSD between any two CA traces corresponding to two individuals in a generation.

The bottom panel of S6 Fig shows the expected drop-off in diversity per generation when the different neighborhood sizes are employed. As expected, when employing the global selection operator, the diversity drops sharply very early on, as SIfTER is converging prematurely to a few local minima. The other neighborhood sizes provide a much more gradual loss in diversity and converge overall to much higher diversity. $C25$ provides a good compromise, and is the setting we employ to obtain the landscapes analyzed in the Results section. It is worth noting that in addition to allowing rigorous determination of the neighborhood size parameter, the analysis shown in the bottom panel of S6 Fig additionally points out that convergence is reached by generation 50. Hence, any number of generations no smaller than this value is sufficient to allow SIfTER to explore the breadth of the conformation space.

Analysis on Robustness of SIfTER To demonstrate robustness of results obtained by SIfTER we show results obtained from three independent runs of the algorithm. S7 Fig juxtapose landscapes obtained for H-Ras WT from three different runs and superimposes distributions of energies of functional conformations (with Rosetta score12 below the -100 threshold) obtained from the three runs. Run #1 corresponds to the results analyzed in the manuscript. The histograms shown in the bottom panel are obtained via kernel density estimation in R. S7 Fig shows that the landscapes and the distributions of energies are nearly identical. Taken together, this analysis demonstrates that the algorithm is robust and reliable.

Analysis on Value of Additional Populations in SIfTER Here we justify the need for further exploration through additional populations in SIfTER as opposed to the initial population only. S8 Fig shows the energy landscape associated with functional conformations (as defined in the main text) generated by SIfTER for WT H-Ras after all data is compiled together at the end of its 100 generations. The conformations of the initial population are superimposed over the landscape. These conformations are color-coded according to their energetic difference from the lowest-energy conformation among the functional conformations generated by SIfTER from all its generations. S8 Fig shows that additional populations in SIfTER are needed to fill in regions of the conformation space (and associated energy landscape) not covered by either

the crystallographic structures or the additional ones obtained by perturbing them in the initial population; that is, a simple procedure that interpolates over crystallographic structures and even applies the local improvement operator to conformations resulting from the interpolations would miss important regions of the energy landscape. This is particularly appreciated when considering that the true search space is of 10 dimensions rather than the 2 used to visualize the energy landscape. These results justify running SIfTERfor more than just the initial population. Indeed, many of the early generations in SIfTERare responsible for exploring new regions of the conformation space, whereas the latter ones are responsible for driving deeper into explored regions and thus mapping out the basins in the landscape. A dynamic picture of SIfTERin action can be seen in the animation provided in the following link: http:`http://cs.gmu.edu/~ashehu/sites/default/files/tools/SIfTER_PCB_2015/RasWT_genMovie.mp4`

Visualizing Projections Along PC3 We provide more detail and show projections of the energy surface along PC3, as well. S9 Fig does so for each of the three sequences, showing projections on PC1 and PC3 and then on P2 and PC3. As S9 Fig shows, retaining PC1 is crucial in order to visualize both the On and Off basins. In particular, removal of PC1, as can be seen in the projections along PC2 and PC3 alone, removes the distinction between the On and Off basins; the two are merged in one. So, PC1 is crucial to maintain as a projection axis for visualization of the energy landscapes. Moreover, if the projection was limited to PC1 and PC3 instead of PC1 and PC2, the distinction between the other two basins, Conf1 and Conf2, would be lost. So, taken together, S9 Fig makes the case that projecting along PC1 and PC2 does not hide any details, nor do the other projections introduce any states not observed by the PC1-PC2 landscapes.

Energetic Variance Analysis S10 Fig shows the variance of the energy values behind each cell in the grid imposed over PC1 and PC2 for visualization of the energy landscapes; instead of color-coding each cell according to the median value over energies of conformations mapping to it, the variance is used instead. This is done for each of the three sequences, limited to the Rosetta energy values. S10 Fig shows lower variance for the four structural states/basins; the algorithm explores these in greater structural detail, as it is driven towards lower-energy regions of the search space in its optimization process. The On and Off basins are clearly distinct, as seen in the obtained WT H-Ras landscape. The median energy in the On basin (a region conservatively defined with 5 ¡ PC1 ¡ 10 and -20 ¡ PC2 ¡ 2.5) is -344 score12 units, whereas the median energy in the Off basin (a region conservatively defined with -20 ¡ PC1 ¡ -12.5 and -7.5 ¡ PC2 ¡ 0) is -280 score12 units. In contrast, as the WT H-Ras energy landscape shows, there are higher-energy structures separating the On and Off basin, and the energies of these structures go from -225 to -150. The juxtaposition of representative structures from each of the four identified basins in Fig. 6 in the manuscript also clearly shows that there are structural differences between the

four captured structural states.

References

1. Bradley P, Misura KM, Baker D. Toward high-resolution de novo structure prediction for small proteins. Science 2005;309(5742):1868–1871.
