# Supplementary material for: Mapping the Conformation Space of Wildtype and Mutant H-Ras with a Memetic, Cellular, and Multiscale Evolutionary Algorithm
Source: PLoS Comput Biol. 2015 Sep 1;11(9):e1004470. doi: 10.1371/journal.pcbi.1004470 (PMC4556523; doi:10.1371/journal.pcbi.1004470)
Supplement: S1 Table — The list of PDB ids corresponding to crystallographic structures extracted from the PDB for H-Ras (WT and variants) is shown. Structures used by the PCA are labeled either GTP or GDP. Structures withheld from the PCA but used for validation are labeled Validation. (PDF) [file pcbi.1004470.s002.pdf]

Table 1: **List of PDB Ids of Crystallographic Structures.**

Nr.	PDB ID	Chain	Label	Nr.	PDB ID	Chain	Label
1	1lf5	A	GDP	44	6q21	D	GTP
2	1q21	A	GDP	45	721p	A	GTP
3	1xj0	A	GDP	46	821p	A	GTP
4	1zvq	A	GDP	47	1BKD	R	Validation
5	2ce2	X	GDP	48	2CL6	X	Validation
6	2q21	A	GDP	49	2QUZ	A	Validation
7	4q21	A	GDP	50	2RGA	A	Validation
8	121p	A	GTP	51	2RGB	A	Validation
9	1agp	A	GTP	52	2RGC	A	Validation
10	1ctq	A	GTP	53	2RGD	A	Validation
11	1gnp	A	GTP	54	2UZI	R	Validation
12	1gnq	A	GTP	55	2VH5	R	Validation
13	1gnr	A	GTP	56	3DDC	A	Validation
14	1he8	B	GTP	57	3I3S	R	Validation
15	1jah	A	GTP	58	3K8Y	A	Validation
16	1jai	A	GTP	59	3KKN	A	Validation
17	1k8r	A	GTP	60	3L8Y	A	Validation
18	1lf0	A	GTP	61	3L8Z	A	Validation
19	1lfd	B	GTP	62	3LBH	A	Validation
20	1lfd	D	GTP	63	3LBI	A	Validation
21	1nvu	Q	GTP	64	3LBN	A	Validation
22	1nvv	Q	GTP	65	3OIU	A	Validation
23	1nvw	Q	GTP	66	3OIW	A	Validation
24	1nvx	Q	GTP	67	3RRY	A	Validation
25	1p2s	A	GTP	68	3RRZ	A	Validation
26	1p2v	A	GTP	69	3RS0	A	Validation
27	1qra	A	GTP	70	3RS2	A	Validation
28	1wq1	R	GTP	71	3RS3	A	Validation
29	1xd2	A	GTP	72	3RS5	A	Validation
30	221p	A	GTP	73	3RSO	A	Validation
31	2c5l	A	GTP	74	3TGP	A	Validation
32	2c5l	B	GTP	75	4DLR	A	Validation
33	2cl0	X	GTP	76	4DLS	A	Validation
34	2cl7	X	GTP	77	4DLT	A	Validation
35	2clc	X	GTP	78	4DLU	A	Validation
36	2evw	X	GTP	79	4DLV	A	Validation
37	421p	A	GTP	80	4DLW	A	Validation
38	521p	A	GTP	81	4DLX	A	Validation
39	5p21	A	GTP	82	4DLY	A	Validation
40	621p	A	GTP	83	4DLZ	A	Validation
41	6q21	A	GTP	84	4EFL	A	Validation
42	6q21	B	GTP	85	4EFM	A	Validation
43	6q21	C	GTP	86	4EFN	A	Validation
