# Supplementary material for: Mapping the Conformation Space of Wildtype and Mutant H-Ras with a Memetic, Cellular, and Multiscale Evolutionary Algorithm
Source: PLoS Comput Biol. 2015 Sep 1;11(9):e1004470. doi: 10.1371/journal.pcbi.1004470 (PMC4556523; doi:10.1371/journal.pcbi.1004470)
Supplement: S2 Table — PDB ids of 5 crystallographic structures deemed outliers are listed here, together with information on the papers introducing them to show the two labs contributing them to the PDB. (PDF) [file pcbi.1004470.s003.pdf]

Table 1: **Details on Crystallographic Structures Deemed Outliers.**

**PDB ID**	**Title**	**Authors**
4EFM, 4EFL, 4EFN	Crystal structures of the state1 conformations of the GTP-bound H-Ras protein and its oncogenic G12V and Q61L mutants [**?**]	Kataoka lab 2012
3KKN	Structural basis for conformational dynamics of GTP-bound Ras protein [**?**]	Kataoka lab 2010
1BKD	The structural basis of the activation of Ras by Sos [**?**]	Kuriyan lab 1998
