# Supplementary figures and images for: Mapping the Conformation Space of Wildtype and Mutant H-Ras with a Memetic, Cellular, and Multiscale Evolutionary Algorithm
Source: PLoS Comput Biol. 2015 Sep 1;11(9):e1004470. doi: 10.1371/journal.pcbi.1004470 (PMC4556523; doi:10.1371/journal.pcbi.1004470)

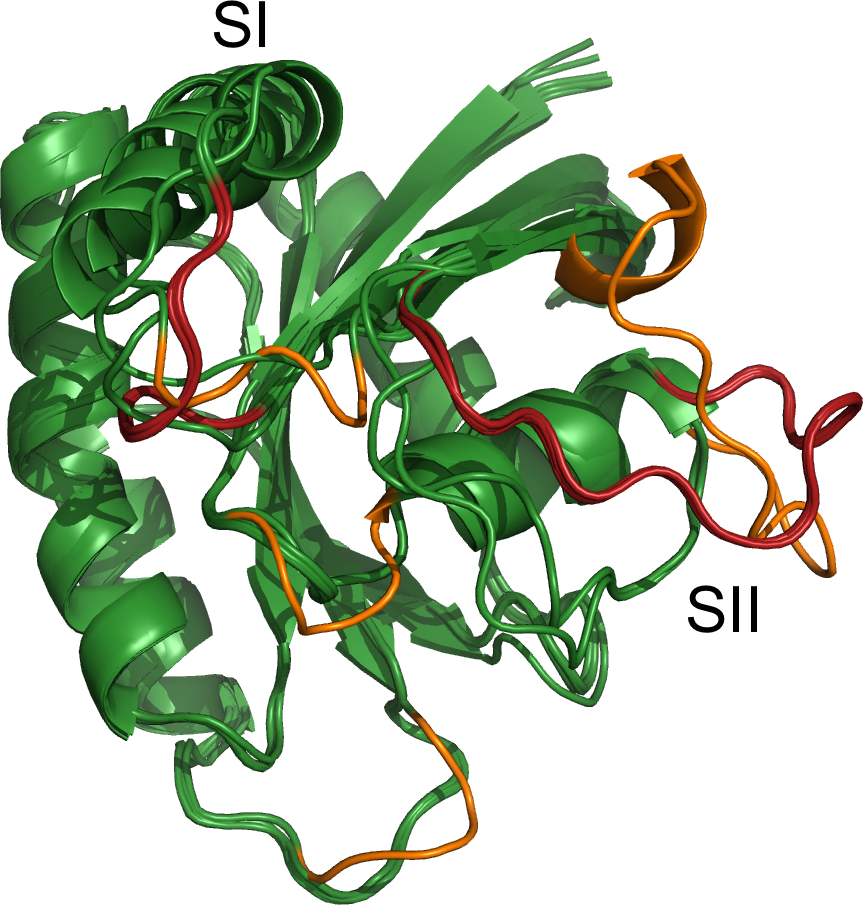

Supplement: S1 Fig — The 5 crystallographic structures deemed outliers are shown here in red (PDB ids 4EFM, 4EFL, 4EFN, 3KKN) and orange (PDB id 1BKD), superimposed over a representative structure (drawn in green). The SI and SII regions are denoted. (TIFF) [file pcbi.1004470.s004.tiff]

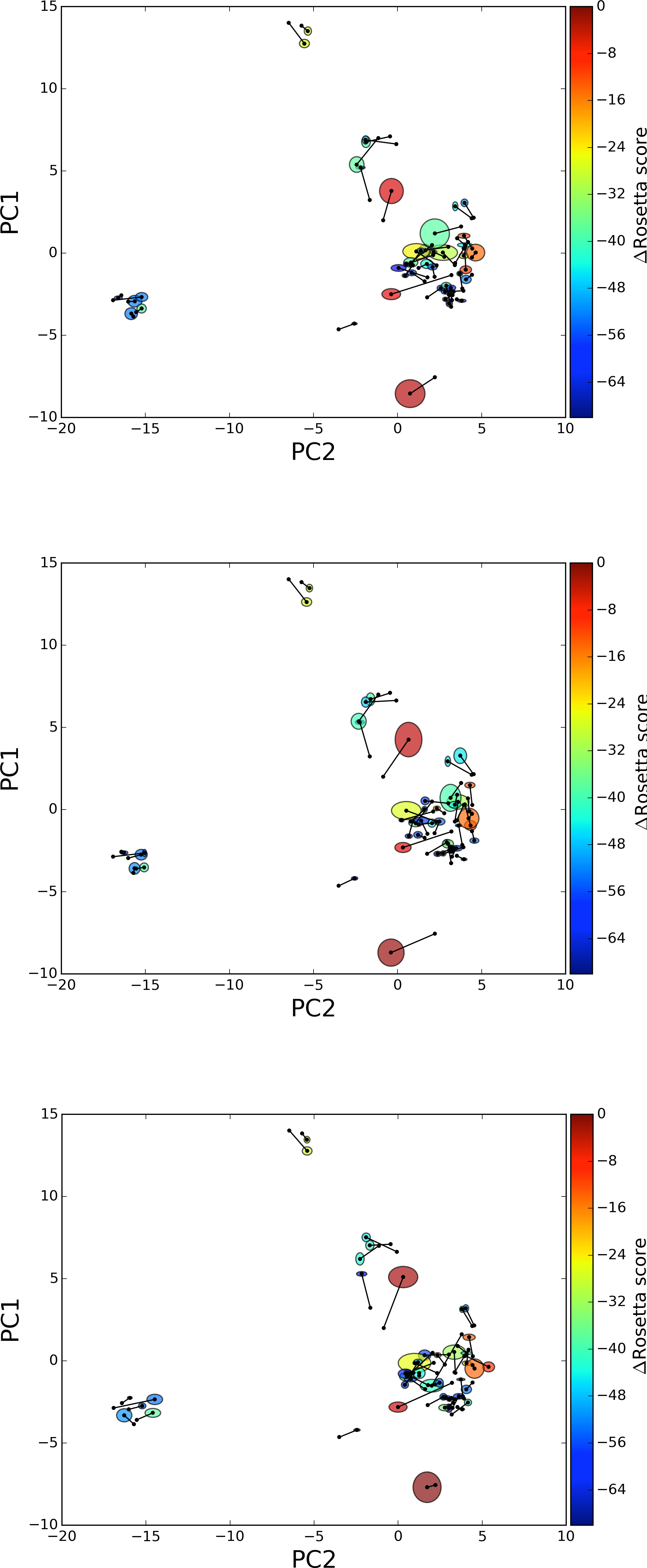

Supplement: S4 Fig — Magnitude and direction along which the Rosetta energy function wants to move crystallographic structures in the score12 all-atom landscape are shown here. The process is repeated for each sequence of H-Ras considered here, with the WT shown in the top panel, G12V in the middle panel, and Q61L in the bottom panel. (TIFF) [file pcbi.1004470.s007.tiff]

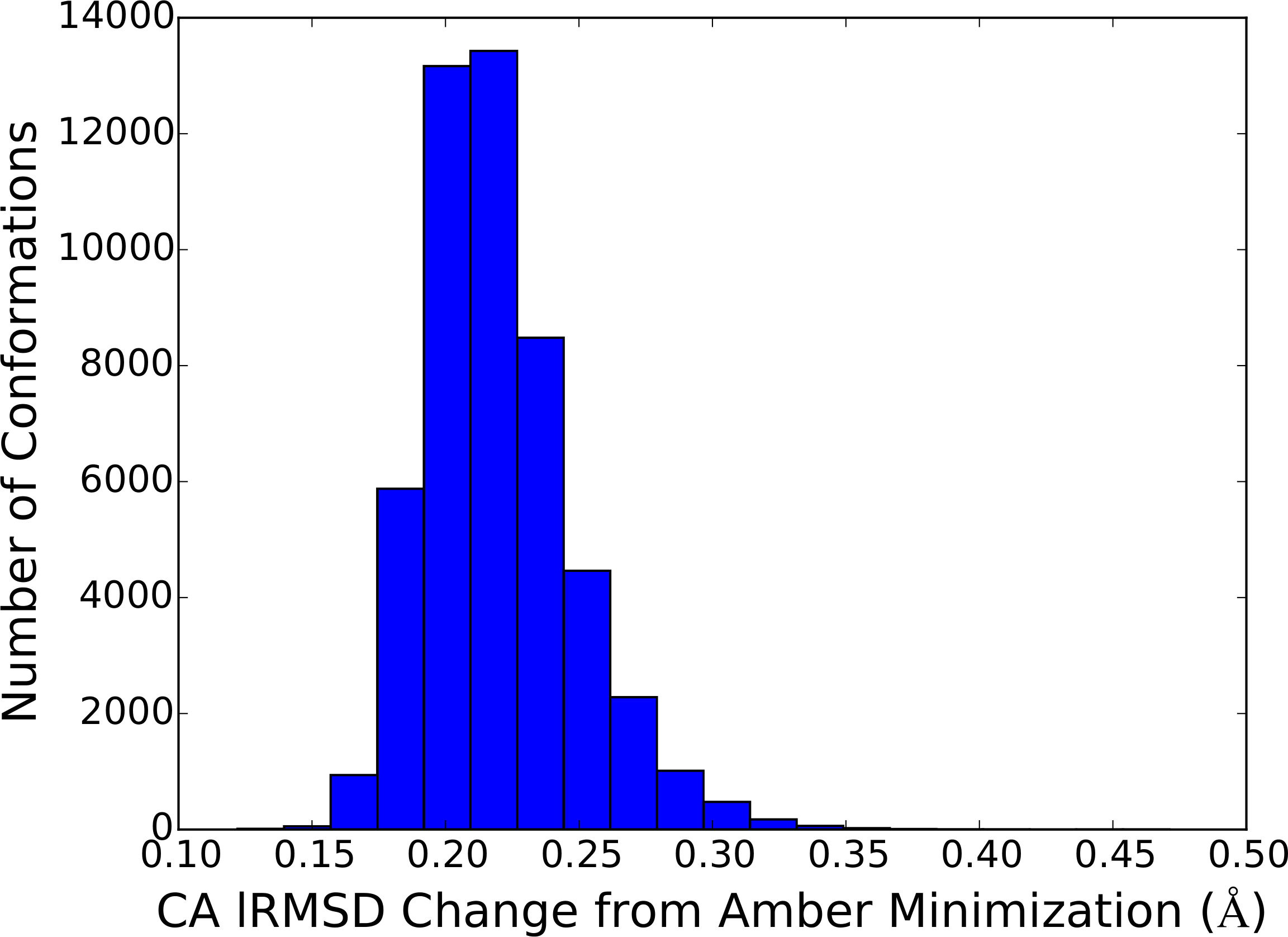

Supplement: S5 Fig — Change due to Amber minimization protocol is shown here for all functional conformations obtained by SIfTER for WT H-Ras in terms of CA RMSD. (TIFF) [file pcbi.1004470.s008.tiff]

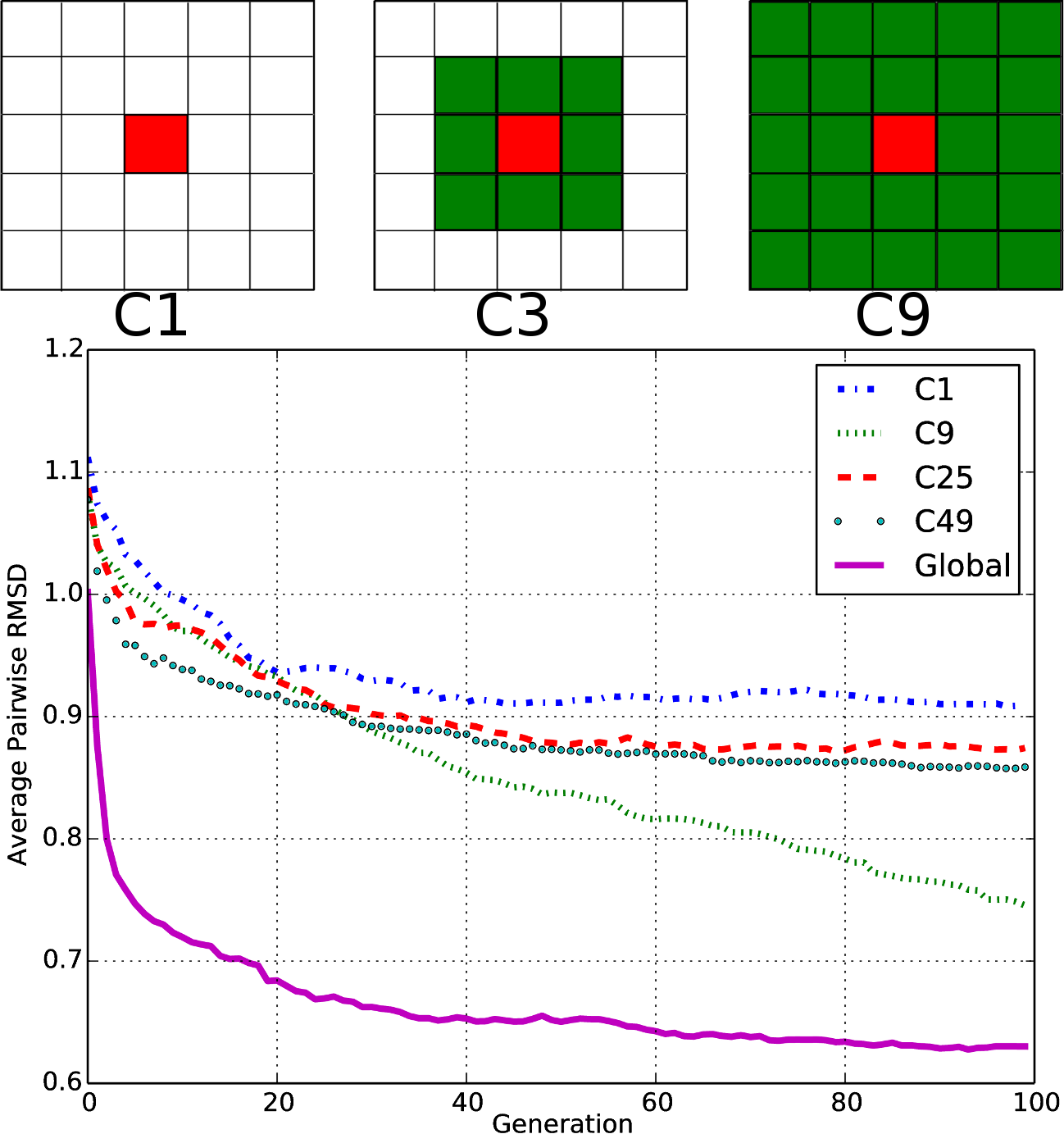

Supplement: S6 Fig — Top panel: The C1 (left), C9 (middle), and C25 (right) neighborhoods are illustrated here. The cell populated by the offspring is drawn in red. Cells in green are additional neighboring cells considered by the local selection operator when increasing the C parameter. Bottom panel: The structural diversity of the population in each generation is tracked across 100 generations. This is done for five settings of C in the local selection operator: C1, C9, C25, C49, and C∞. The latter corresponds to global selection. (TIFF) [file pcbi.1004470.s009.tiff]

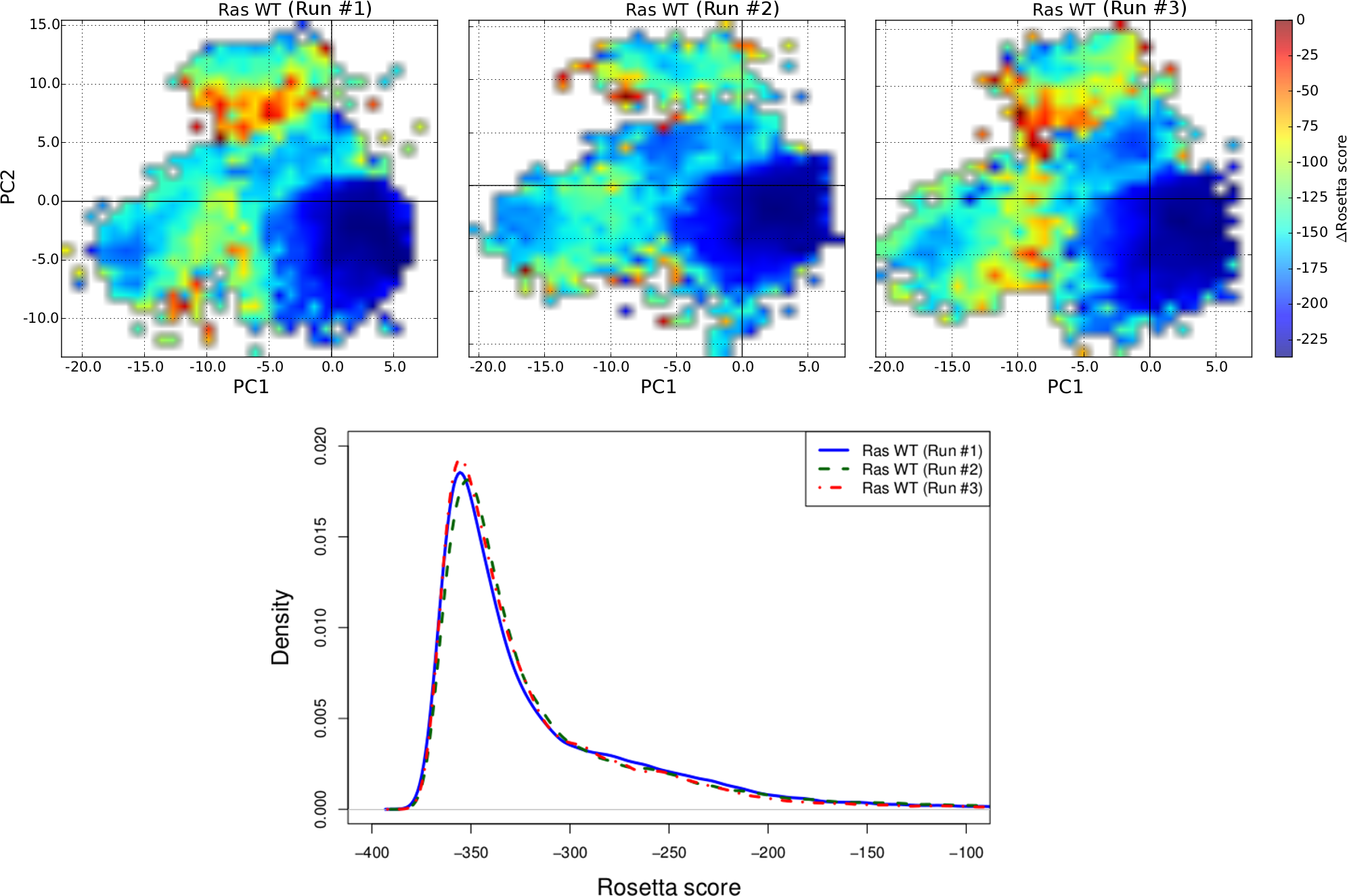

Supplement: S7 Fig — Top panel: Three landscapes are shown here for H-Ras WT obtained from three different runs of SIfTER Bottom panel: Distributions of energies obtained on the H-Ras WT from three different runs of SIfTER are superimposed over one another. (TIFF) [file pcbi.1004470.s010.tiff]

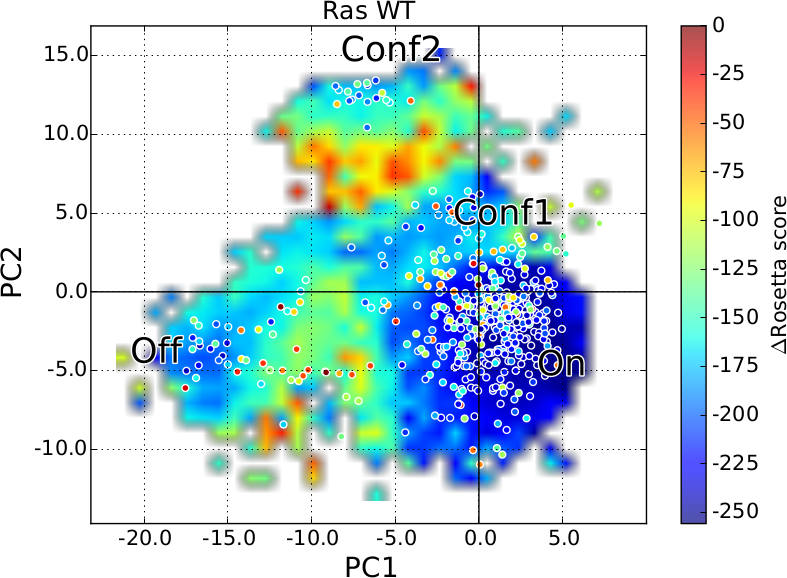

Supplement: S8 Fig — The energy landscape associated with functional conformations generated by SIfTER for WT H-Ras is shown here, together with the conformations of the initial population. The latter are color-coded according to their energetic difference from the lowest-energy conformation among the functional conformations. It can be seen that additional populations in SIfTER are needed to fill in regions of the conformation space (and associated energy landscape) not covered by either the crystallographic structures or the additional ones obtained by perturbing them in the initial population. (TIFF) [file pcbi.1004470.s011.tiff]

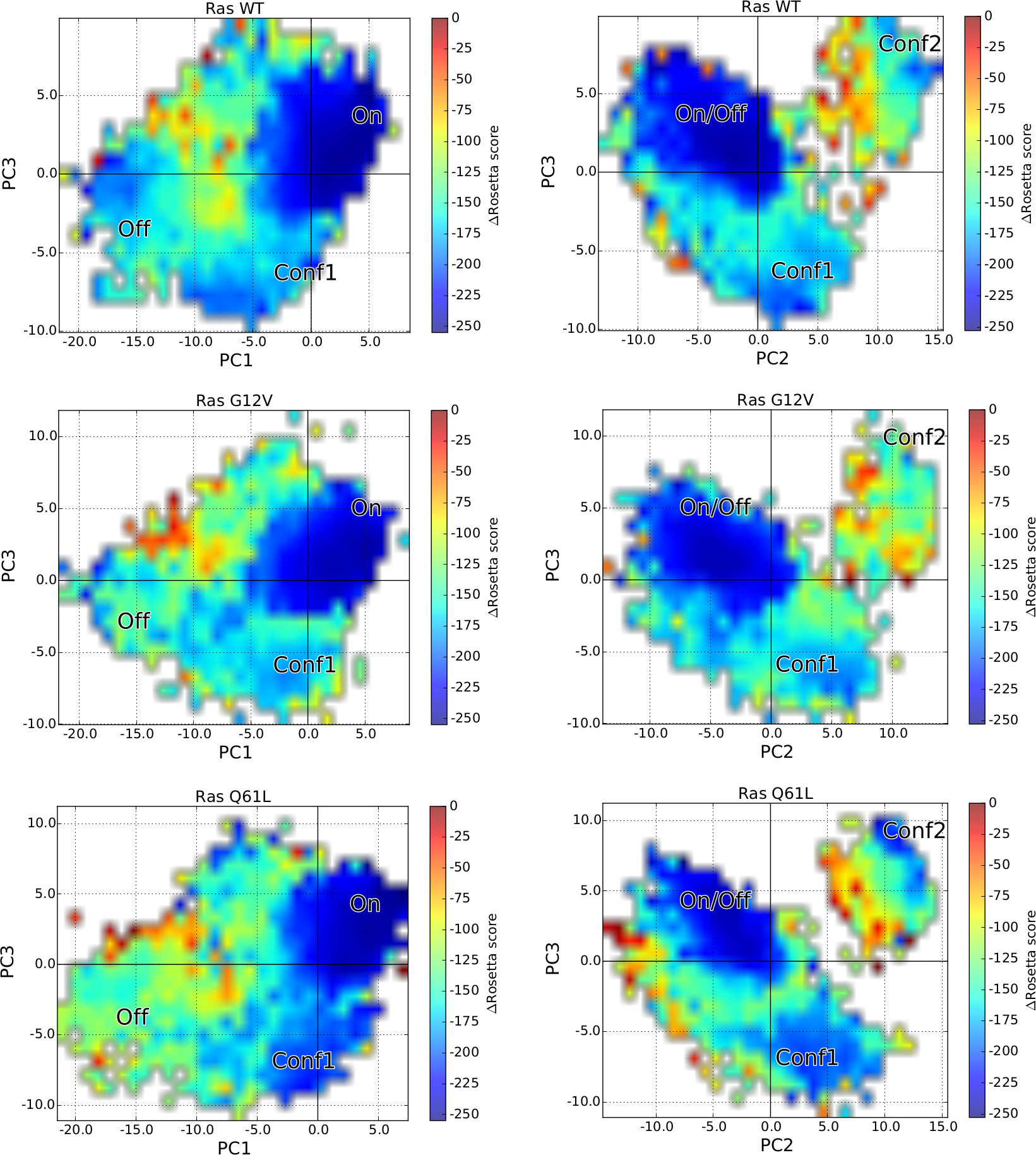

Supplement: S9 Fig — Projections are shown along PC3, as well, for each of the three sequences. The color-coding is as described in the manuscript. The states are labeled to the extent that they are visible along PC3. (TIFF) [file pcbi.1004470.s012.tiff]

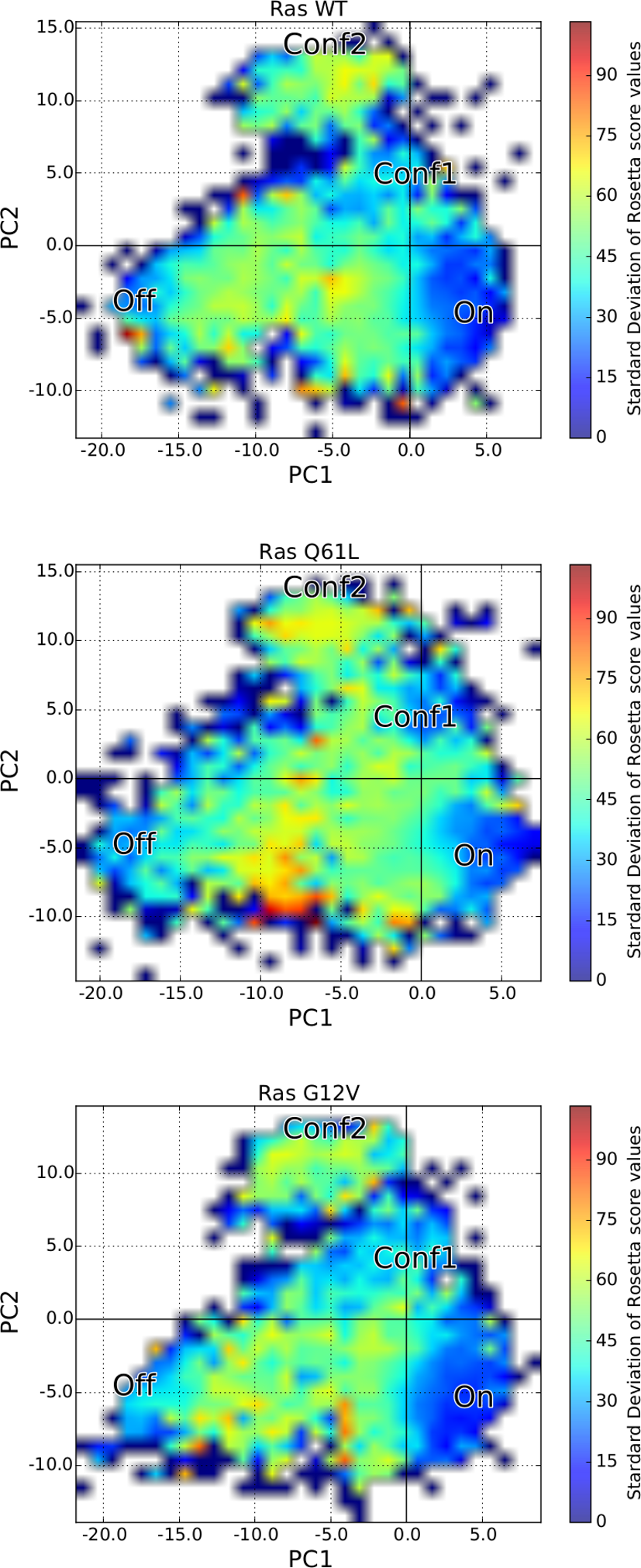

Supplement: S10 Fig — The variance of the energy values behind each cell in the grid imposed over PC1 and PC2 for visualization of the energy landscapes is shown here as follows: instead of color-coding each cell according to the median value over energies of conformations mapping to it, the variance is used instead. This is done for each of the three sequences. (TIFF) [file pcbi.1004470.s013.tiff]
